# The effects of interacting with fish in aquariums on human health and well-being: A systematic review

**DOI:** 10.1371/journal.pone.0220524

**Published:** 2019-07-29

**Authors:** Heather Clements, Stephanie Valentin, Nicholas Jenkins, Jean Rankin, Julien S. Baker, Nancy Gee, Donna Snellgrove, Katherine Sloman

**Affiliations:** 1 Institute of Biomedical and Environmental Health Research, School of Health and Life Sciences, University of the West of Scotland, Paisley, United Kingdom; 2 Institute of Clinical Exercise and Health Science, School of Health and Life Sciences, University of the West of Scotland, Lanarkshire, United Kingdom; 3 School of Media, Culture and Society, University of the West of Scotland, Lanarkshire, United Kingdom; 4 School of Health and Life Sciences, University of the West of Scotland, Paisley, United Kingdom; 5 WALTHAM Centre for Pet Nutrition, Leicestershire, United Kingdom; 6 Department of Psychology, State University of New York, Fredonia, New York, United States of America; Jagiellonian University Medical College, POLAND

## Abstract

**Background:**

Most research into the health benefits of human-animal interaction has focused on species that interact physically with humans, such as dogs. This may be unsuitable for certain populations for reasons including accessibility and the risk of negative consequences to both the person and the animal. However, some research has associated viewing fish in aquariums with positive well-being outcomes; as there is no physical contact with the animal, this form of interaction carries less risk. At present, little is known about the specific benefits of human-fish interaction.

**Objectives:**

To explore current evidence relating to the psychological and physiological benefits of interacting with fish in aquariums.

**Methods:**

Systematic searches were conducted to identify relevant primary research of any design. All forms of interaction were considered, including keeping fish as companion animals and fish aquarium-based interventions. “Non-live” alternatives, such as videos, were also considered. This review was conducted according to a registered protocol (PROSPERO ID: CRD42018090466).

**Results:**

Nineteen studies were included. Two provided tentative evidence that keeping home aquaria is associated with relaxation. The remaining studies involved novel interactions with fish in home or public aquariums. Outcomes relating to anxiety, relaxation and/or physiological stress were commonly assessed; evidence was mixed with both positive and null findings. Preliminary support was found for effects on mood, pain, nutritional intake and body weight, but not loneliness. All studies had methodological issues and risk of bias was either high or unclear.

**Conclusions:**

Review findings suggest that interacting with fish in aquariums has the potential to benefit human well-being, although research on this topic is currently limited. Future research should aim to address gaps in the evidence, such as whether and how the type of human-fish interaction can influence well-being outcomes. Researchers should also aim to address the methodological concerns highlighted in this review.

## Introduction

Interacting with non-human animals (hereafter “animals”) has been associated with a range of well-being benefits among humans. Companion animal guardianship has been linked with improved physical and psychological outcomes, including lower blood pressure [1,2], reduced risk of cardiovascular disease and lower rates of mortality [3,4], reduced loneliness [5], and increased emotional support during mental health crisis [6]. In fact, research has indicated that many people choose to keep companion animals for reasons associated with well-being, such as companionship, emotional support, and improved physical health [7,8]. Similarly, animal-assisted interventions (AAI) are initiated with the specific purpose of improving one or more aspects of human well-being; they include goal-oriented animal-assisted therapies delivered by healthcare professionals, and animal-assisted activities which are often volunteer-led and may lack specific treatment goals [9]. These interventions have been used to support improvements in physical, psychological, and behavioural outcomes for a wide range of populations across the lifespan [10–14].

Despite these positive findings, research into the benefits of human-animal interaction (HAI) is far from conclusive. Some studies have shown no relationship, or a negative relationship, between keeping companion animals and physical or mental health outcomes [15–20]. Furthermore, as most research in this area is correlational it is difficult to determine causality; better health may increase the likelihood of adopting a companion animal, rather than the reverse [21], or health and companion animal guardianship may be linked by other factors, such as sociodemographic characteristics or health-related behaviours [19,22,23]. Similarly, while AAI are commonly perceived as beneficial, especially among those with a positive attitude towards companion animals, some authors suggest that these benefits have been overstated [24]. In reality, research concerning these interventions is frequently anecdotal or descriptive in nature, with high levels of heterogeneity in factors such as the type of animal, the nature of the interaction, and the setting [25,26]. Methodological issues are also commonplace and include the absence of appropriate comparison groups, reliance on small samples, failure to randomise participants to conditions, and a lack of blinding for both participants and assessors [25–28]. It is therefore difficult to draw firm conclusions about the efficacy of these interventions [26–28].

These inconsistencies are further confounded by a lack of consensus about the mechanisms through which HAI may improve human well-being [9]. Researchers have often referred to the biophilia hypothesis [29,30], which proposes that humans have an innate affiliation with other forms of life. This perspective suggests that because human evolution occurred almost exclusively in natural environments, people are predisposed to respond positively to aspects of nature that would have increased fitness in the ancestral environment, and negatively to those which would have decreased fitness [31]. For example, people typically respond positively to natural landscapes providing sources of food, water or shelter, and negatively to animals which pose a threat, such as spiders or snakes [31]. Although the biophilia hypothesis has been criticised for offering too broad a perspective and for lacking falsifiability [32], researchers have drawn on these ideas to develop theories with more explanatory power. For example, the biophilia-effect suggests that because the behaviour of animals is indicative of the presence or absence of threats in the environment, interaction with a calm or friendly animal may support human well-being by promoting relaxation and reducing physiological arousal [33,34].

Other popular explanations centre on the social support provided by companion animals. In the context of attachment theory [35–37] for example, humans are argued to form bonds with their companion animals which are comparable to those formed within close interpersonal relationships [38]. This theory suggests that humans form strong emotional attachments with certain individuals, or “attachment figures”. These attachments are characterised by the presence of proximity seeking behaviours, distress at separation, and the provision of unique emotional support that cannot be replicated within other interpersonal relationships. Although attachment theory originally focused on the relationship between an infant and their primary caregiver (usually their mother), it was later expanded to incorporate the bonds which form in other close relationships, such as with siblings or romantic partners [39,40]. More recently, attachment theory has been applied to human-animal relationships, with findings suggesting that both the human and animal can serve as the attachment figure and provide feelings of comfort and safety during times of uncertainty or stress [20,38]. Furthermore, support provided by animals may be particularly effective, as it is unconditional and non-judgemental [41], and because physical touch–an important component of emotional support–is often discouraged with other humans but not with animals [42].

Alternatively, HAI may operate via distraction, whereby attention is diverted away from a perceived stressor to lessen the experience of negative mental states; this may be of most relevance in the context of AAI [25]. Research has indicated that young children preferentially attend to images or videos of animals compared to non-living objects [43], and will choose to interact with real (but caged) animals over toys resembling those animals [44]. Similarly, adults have been found to more rapidly identify changes in the location of living targets (animals and people), compared to inanimate objects [45]. These findings suggest that animals may be particularly effective at attracting human attention. However, animals are not unique in being an effective source of distraction, and so similar benefits may be achieved through the use of alternative, and possibly more cost effective, stimuli [25,42].

This brief overview is by no means exhaustive and several other theories have been proposed [9,33,42]. Despite these divergent approaches however, one model provides a framework which may potentially incorporate some, or all, of the above discussed mechanisms. The biopsychosocial model [46] proposes that health is a continuum influenced by interacting biological, psychological and social factors; changes in one factor may influence the others and in turn impact health. For example, psychosocial stresses may lead to physiological responses including increased heart rate and blood pressure, or reduced immune function. Ultimately, these responses may have a negative effect on health, resulting in increased morbidity and mortality [47,48]. Equally however, some psychological and social factors may have a protective influence on health; higher levels of social support for example, have been linked to a reduced risk of cardiovascular disease [47]. In the context of this model, there are numerous ways in which interaction with companion animals may impact human health. For example, owning a dog may lead to improved health through increased physical activity, while animals may provide social support either directly, or indirectly by facilitating social interactions with other individuals [49,50]. Conversely however, the grief associated with the loss of a companion animal may have a detrimental effect on human well-being [50]. Thus, while the biopsychosocial model provides a potential framework for integrating multiple theories, it also highlights the likelihood that no one mechanism can account for the diverse effects of HAI. One area which warrants further consideration is whether the observed benefits are influenced by the type of animal involved in the interaction [26,51].

Multiple reviews and meta-analyses have noted that dogs are the animal most frequently involved in AAI (although other species such as horses are also commonly involved) [10,12,51]. Similarly, much research into companion animals has focused on those animals that can interact physically with humans, such as dogs and cats [3,52]. This type of interaction may not however, be suitable among all populations. For instance, people in rented accommodation are often restricted in the types of companion animal they may keep in their home, and physical interactions may be inappropriate for people with declining health or limited physical capacity [52]. Similarly, dog-assisted (or similar) interventions often rely on volunteer services [51] and may require supervision of the client and animal to minimise risk, which can lead to infrequent and inconsistent exposure [53,54]. Issues may also arise where there is potential for aggression from the animal, where individuals have allergies, compromised immune systems, or phobias, or where contact with the animal could lead to accidental injury (e.g. scratches, falls) [51,55,56]. Animal welfare is also a concern, as some clients may behave aggressively or unpredictably towards the animal, or the animal may become stressed during the interaction [51,57]. Therefore, research into the effects of HAI with less physically interactive animals is needed to determine whether benefits may be experienced.

One form of HAI which has attracted relatively little investigation is the role of fish aquariums. Early research indicated a link between viewing fish in aquariums and benefits such as reduced blood pressure and increased relaxation [58–60], perhaps contributing to the widespread notion that aquariums are beneficial in healthcare settings [61]. More recently, research has linked interaction with fish in aquariums to outcomes such as reduced anxiety [62], increased tolerance to pain [63], and improvements in nutritional intake and body weight among residents of specialised dementia units [64,65]. As with HAI research more broadly, the mechanisms underlying these benefits are unclear. Research with people who keep home aquaria has indicated that some individuals consider their fish to be a source of companionship, and feel an emotional bond with the animals [52]; this suggests social support and attachment may play a role in the beneficial effects of human-fish interaction. However, while research has shown the presence of attachment behaviours in other human-animal relationships, such as with dogs [66,67], it is not evident that fish exhibit behaviours such as proximity seeking or separation distress. Thus, while individuals may believe there to be an emotional bond between themselves and their fish, it is unclear whether this constitutes a true attachment bond as described by attachment theory [35–37]. Alternatively, watching fish swimming may simply be a source of distraction; this is supported by research which has shown positive physiological effects associated with viewing videos of animals, including fish [68].

An alternative perspective still comes from theories concerning the restorative value of nature. These theories suggest that exposure to unthreatening nature can help restore depleted cognitive resources, and support rapid emotional and physiological recovery from stressful events [69]. Although most of this research has focused on natural or “green” landscapes, some studies have directly explored the role that encounters with wildlife play in human well-being. For example, research has suggested that many people feed wild birds because doing so brings them pleasure [70], while watching wild birds feeding is associated with increased relaxation and connectedness to nature [71]. Similarly, improvements in self-reported control, happiness, and activity were observed among a sample of nursing home residents who were given the responsibility of caring for a bird feeder, while no changes were observed among residents who did not receive such an opportunity [72]. Furthermore, research has suggested that for some individuals, wildlife encounters are a key motivation for visiting natural (coastal) environments [73], and are associated with a range of benefits to human psychological well-being [74]. Given that the ways in which people interact with birds and other forms of wildlife are similar to the ways in which people interact with fish in aquariums (i.e. the interaction is largely visual), it may be that watching fish swimming promotes human well-being because this activity provides exposure to unthreatening nature, leading to restoration.

Irrespective of the mechanism, these findings suggest that human-fish interactions may be a viable alternative to more commonly researched forms of HAI. Furthermore, aquariums may overcome some of the issues associated with these forms of interaction. As a constant feature within the environment, fish aquariums are available to the client at any time and for as long as required, thus may provide greater flexibility in exposure than AAI which rely on visitation programmes [53]. Even other types of resident animal cannot provide constant interaction, as this would be detrimental to their welfare [51]. The monetary cost associated with installation and upkeep of a fish tank is also much smaller than that associated with other companion animals [51], although regular maintenance of the aquarium is needed, and requires an individual with knowledge of the necessary processes to ensure the welfare of the fish is not compromised. Aside from the person responsible for maintaining the fish tanks however, the passive nature of viewing fish in an aquarium means that even individuals with limited physical capacity are able to interact with the animals [52]. There are no significant risks from aggression or allergies, and fewer risks associated with accidental injury due to the lack of physical contact with the animal (although possible injury could be sustained while installing or maintaining the tank, or if someone or something damages the tank, causing a break). While there is a small risk of bacterial infection associated with keeping home aquaria, this is rare and requires physical contact with the fish or water, so can be effectively minimised through careful hygiene practices [75].

Despite the potential benefits however, research in this area is limited, and to date only one review has sought to explore the potential benefits of fish aquariums to human health and well-being [76]. However, this narrative review explored these benefits in the context of restorative environments and biodiversity, with a focus on the value of public aquariums; although there was reference to research conducted with home aquaria, this overview was not comprehensive. Furthermore, consideration should be given to the quality and strength of evidence when drawing conclusions from existing research findings; for this purpose, a systematic review of the literature is needed.

### Review questions

Through a systematic review of the literature, this article aims to explore the psychological and physiological benefits of interacting with fish in aquariums. Given that previous research has highlighted potential benefits associated with viewing videos of fish [68], simulated or “non-live” alternatives will also be considered. The following research questions will be addressed:

1. What influence does interaction with fish in aquariums (live or non-live) have on the psychological well-being of human participants?
2. What influence does interaction with fish in aquariums (live or non-live) have on the physiological well-being of human participants?

In addition, this review will aim to identify:

- The attitudes of human participants regarding the benefits and challenges of interacting with fish in aquariums;
- Any adverse effects which may be experienced by humans when interacting with fish in aquariums;
- Any concerns regarding animal welfare which may be encountered during human interaction with fish in aquariums.

## Methods

This systematic review was conducted according to a registered protocol (PROSPERO ID: CRD42018090466), and adhered to the Preferred Reporting Items for Systematic Reviews and Meta-analysis (PRISMA) statements [77]. Prior to commencing the review, the PROSPERO register was searched to ensure no similar reviews were currently underway; the following terms were used: “fish”, “aquarium”, “animal assisted intervention”, “animal assisted therapy”, “pet therapy”, “human-animal interaction” and “companion animal”.

### Search strategy

Systematic searches conducted in January 2018 identified peer-reviewed evidence and grey literature on the topic of fish aquarium-based HAI. A four-step search strategy was developed through discussion between the authors, and consulting previous systematic reviews in the field of HAI. Searches were conducted in the following electronic databases: Cumulative Index to Nursing and Allied Health Literature (CINAHL), Education Source, ERIC, Health Source–Nursing/Academic Edition, MEDLINE, PsycARTICLES, Psychology and Behavioural Sciences Collection, PubMed, SAGE Journals ONLINE, Science Direct, and Web of Science (Core Collection). Steps one to three involved identifying all records relating to 1) HAI and related theories, 2) relevant health and well-being outcomes, and 3) fish and/or aquariums. Steps one and two were conducted in all fields to maximise identification of relevant literature. However, as much research in this field is conducted with other species, step three was limited to title, abstract and keywords only (or the nearest alternative). Step four combined the results from these searches; an example search strategy is shown in Table 1. Results from electronic databases were supplemented with searches in Google Scholar, EThOS, and websites on HAI (WALTHAM Science, HABRI-Central, and Animals and Society Institute), and by hand-searching the reference lists of included studies and relevant review articles for additional references.

**Table 1:** Example search strategy (PubMed).

Step 1 (all fields):	#1	"human?animal interaction"
	#2	"human?animal relationship"
	#3	"human?animal bond"
	#4	"animal?assisted intervention"
	#5	"animal?assisted therap*"
	#6	"animal?assisted activit*"
	#7	"pet therap*"
	#8	"pet?facilitated therap*"
	#9	"pet?interaction"
	#10	"pet ownership"
	#11	"companion animal"
	#12	attachment
	#13	biophilia
	#14	biopsychosocial
	#15	“social support”
	#16	“social mediation”
	#17	“prepared learning theory”
	#18	“self-efficacy”
	#19	modelling
	#20	“role theory”
	#21	“polyvagal theory”
	#22	“attention restoration theory”
	#23	“stress recovery theory”
	#24	#1 OR #2 OR #3 OR #4 OR #5 OR #6 OR #7 OR #8 OR #9 OR #10 OR #11 OR #12 OR #13 OR #14 OR #15 OR #16 OR #17 OR #18 OR #19 OR #20 OR #21 OR #22 OR #23
Step 2 (all fields):	#25	health
	#26	well?being
	#27	anxiety
	#28	arousal
	#29	stress
	#30	relaxation
	#31	"quality of life"
	#32	"life satisfaction"
	#33	restoration
	#34	recovery
	#35	pain
	#36	loneliness
	#37	#25 OR #26 OR #27 OR #28 OR #29#31 OR #32 OR #33 OR #34 OR #35 OR #36
Step 3 (title/ abstract):	#38	aquarium
	#39	"aquatic environment"
	#40	aquaria
	#41	“fish”
	#42	“fish tank”
	#43	#38 OR #39 OR #40 OR #41 OR #42
Step 4:	#44	#24 AND #37 AND #43

### Inclusion criteria

Based on preliminary searches, research in this area was anticipated to be both limited in quantity and varied in design. Therefore, the inclusion criteria were deliberately broad to incorporate the full scope of research related to effects of interacting with fish in aquariums on human health and well-being. The inclusion criteria were as follows:

*Participants*: there was no limitation on the participant populations of included studies. Studies involving both healthy and clinical samples of any age were included.

*Intervention/exposure*: any form of human interaction with fish in aquariums was included, from passive exposure to fish tanks, actively viewing fish swimming, to caring for fish in aquariums. Non-live alternatives (e.g. videos) were also considered. There were no limitations regarding the length, frequency or duration of exposure, or the setting. Studies were not excluded on the basis that other animals (e.g. corals) were also present in the aquariums.

*Comparator*: studies both with and without a control group were included. For those with a control group, any type of comparator was considered, including no treatment controls, alternative AAI, and alternative interventions without animal involvement.

*Outcomes*: the primary outcomes were psychological (e.g. anxiety, depression, behaviour change, social interaction) and physiological (e.g. blood pressure, heart rate, motor skills) well-being. Secondary outcomes were any adverse events experienced by human participants and any issues regarding animal welfare; participants’ attitudes towards human-fish interaction were also considered, such as any benefits or limitations they had observed, or any evaluations of fish aquarium-based interventions.

*Study Design*: included studies were limited to primary research, but there were no limitations on study design; both quantitative and qualitative studies were included.

### Exclusion criteria

Research was limited to articles published in the English language, with no limitations on date of publication. To enhance the quality of included studies, articles were limited to those published in peer reviewed journals and doctoral theses. Only research involving live fish or non-live alternatives (e.g. videos of fish) was included. Research relating to the health benefits of fish consumption, studies involving invasive research conducted on fish, and those relating to fishing/angling were excluded.

### Study selection

**Fig 1:**
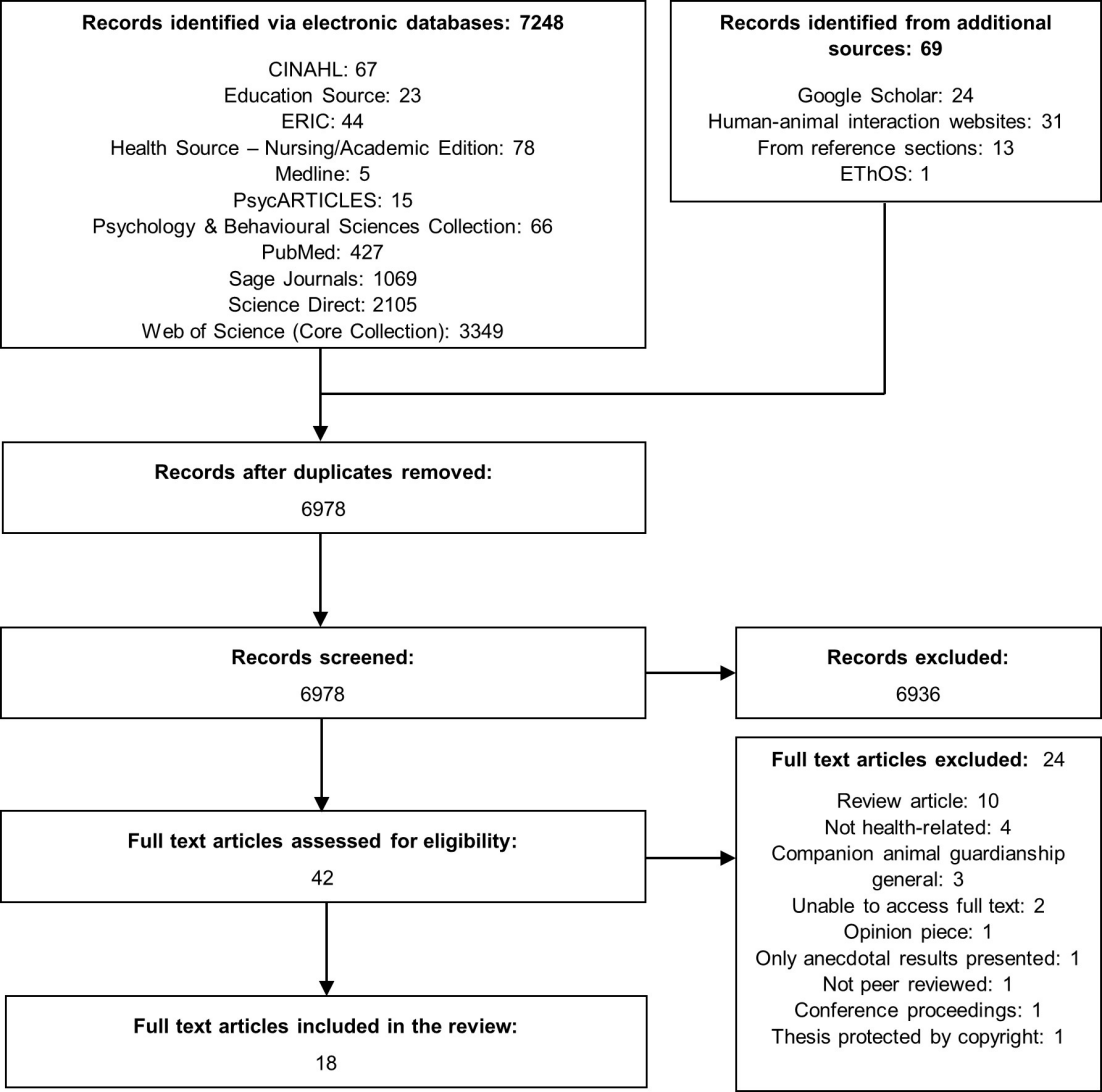
PRISMA flow diagram of study selection.

The study selection process is outlined in Fig 1. All records identified via electronic databases (*n* = 7248) were exported into a single EndNote library and duplicates were removed. All remaining records (*n* = 6978) were then screened for inclusion in a two-stage process. Initially, the titles and abstracts of all records were assessed for relevance by two independent reviewers (HC/KS); the full-text of all remaining articles was then obtained and screened against the inclusion/exclusion criteria by two independent reviewers (HC/SV). At each stage, disagreements were resolved through discussion. Hand-searching of reference lists and supplementary searches on Google Scholar, EThOS, and HAI websites were also conducted to identify any additional studies of relevance (*n* = 69). The full-text of two records, including one unpublished thesis, could not be accessed and attempts to identify up to date contact details for the authors were unsuccessful, so these studies could not be included in the review. A third record (an unpublished thesis) could not be included for copyright reasons, as attempts at gaining the author’s permission to cite the research were unsuccessful.

### Data extraction & quality appraisal

Data from included studies were extracted by two independent reviewers (HC/SV) using a purpose-developed data extraction form (see S3 Appendix). The following data were extracted: general information (author, year, publication type and source, country, funding, conflicts of interest); study details (aims/objectives, dates of data collection, theoretical framework); methods (design, participant recruitment and characteristics, intervention, control, allocation to conditions, setting/context, primary outcomes, secondary outcomes); results (method of analysis, psychological outcomes, physiological outcomes, secondary outcomes); author conclusions; and reviewer comments. All discrepancies were resolved through discussion. To reflect the inclusion of both quantitative and qualitative research, risk of bias was assessed using the National Institute for Health & Care Excellence (NICE) Quality Appraisal Checklists for quantitative intervention studies, quantitative studies reporting a correlation, and qualitative studies [78]. Quality appraisal was conducted by two independent reviewers (HC/SV) and disagreements were resolved through discussion.

### Reporting bias

To assess for selective reporting of outcomes, the methods section of included studies was compared to the presented results to identify any discrepancies and determine whether an adequate description of the results was provided. As no studies were conducted according to a published or registered protocol, it was not possible to draw comparisons between the study protocols and published results. Furthermore, as there was much heterogeneity in study outcomes it was not possible to assess for publication bias using funnel plots, as this is not recommended for use on outcomes assessed in fewer than ten studies [79].

### Strength of evidence

The strength of the evidence was assessed using the Weight of Evidence approach [80] for each of the two review questions independently. This approach involves assessing each study against four criteria. *Weight of Evidence A* is a generic assessment of study quality, while *Weight of Evidence B* and *C* are review-specific, and relate respectively to the appropriateness of the research design and the relevance of the evidence in addressing the review question(s). The final criterion (*Weight of Evidence D*) is an overall assessment of the extent to which the study addresses the review question(s), and was calculated as the most common rating from the first three criteria (where assessments were “low”, “medium” and “high” for the first three criteria, an overall weighting of “medium” was given). Assessments were made independently by two reviewers (HC/SV), with all disagreements resolved through discussion.

## Results

Nineteen studies published in eighteen articles met the inclusion criteria and were included in the review (see Tables 2 and 3 for overview of included studies). All studies were published as peer reviewed journal articles, with publication years ranging from 1984 to 2017. Two articles [54,65] reported research conducted as part of the same project but it is unclear whether the same sample was used for both studies; as the articles reported different outcomes they were treated independently in this review. Most research was conducted in the USA (*n* = 11), with four studies conducted in the UK and one each in Germany, France, Taiwan and Australia.

**Table 2:** Summary of study characteristics of included studies.

First author and year	Design	Participants				Aquarium Intervention(s)	Comparator(s)	Setting (Country)
		Population	Total sample size (*n*)	Age (Mean)	Gender (% female)			
*Fish as companion animals*								
Kidd 1999 [81]	Survey	Pet fish owners	100	37.1 years	50%	-	-	Not applicable (USA)
Langfield 2009 [52]	Phenomenological study, in-depth interviews	Pet fish owners	9	34.9 years	33%	-	-	Not applicable (Australia)
*Correlational studies*								
Lin 2013 [82]	Survey	Medical directors at accredited hospitals	737	49 years	8%	Presence of aquarium in workplace	Presence of other interior amenities (indoor plants; music; art and exhibitions; private or personalised spaces)	Accredited hospitals (Taiwan)
*Intervention studies*								
Barker 2003 [53]	Within-subjects (“crossover”) study	Patients awaiting electroconvulsive therapy treatment	42 (only 30 included in analysis)	48.4 years	74%	10-gallon aquariums containing around five African cichlids, approx. 20-minutes of passive exposure (*n* = 30)	No aquarium (*n* = 30)	Holding/waiting rooms at outpatient treatment centre (USA)
Buttelmann 2014 [62]	Between-subjects study without randomisation	Undergraduate students	71	22.5 years	92%	Five-minute interaction with one veiltail goldfish in 5.5l goldfish bowl, to “try and accustom it to humans” (*n* = 18)	Five-minute interaction with dog (*n* = 18) or plant (*n* = 17), or no activity (*n* = 18)	University laboratory (Germany)
Cole 2000 [83]	Before-and-after pilot study	Patients awaiting heart transplantation	10	55.9 years	20%	15-gallon saltwater tank containing four colourful fish, 11 days (*n* = 10)	-	Hospital rooms (USA)
DeSchriver 1990 [84]	Between-subjects study with randomisation	Older adults in publicly subsidised housing	27	Median per condition = 73–76 years	78%	Eight-minutes viewing 10-gallon tank with nine fish (2 black mollies, 2 red wag swordtails, 2 gold wag moons, 2 pineapple swordtails, 1 catfish) (*n* = 9), or video of tropical fish in an aquarium (*n* = 9)	Eight-minutes viewing “placebo” video of television lines/static (*n* = 9)	Purpose-built laboratory in publicly subsidised housing complex (USA)
Edwards 2002 [64]	Interrupted time-series with non-equivalent control	Residents of specialised dementia units	62	80.1 years	61%	Aquariums containing eight large colourful fish, up to four months (*n* = 62)	Scenic ocean picture, two weeks (*n* = 17)	Dining rooms of specialised dementia units (USA)
Edwards 2013 [65]	Interrupted time-series	Residents of specialised dementia units	70	82.2 years	74%	As above (*n* = 70)	-	As above
Edwards 2014 [54]	Before-and-after study	Residents of specialised dementia units	72	80.3 years	71%	Aquariums containing eight to ten large colourful fish, 10 weeks (*n* = 72)	-	As above
		Staff of specialised dementia units	71	NR	82%	Aquarium as above, 10 weeks (*n* = 71)	-	As above
Katcher 1984 [85]	Between-subjects study with randomisation	Patients undergoing elective dental surgery	42	NR	NR	40-minute contemplation of aquarium with (*n* = 8) or without (*n* = 8) hypnosis	40-minute contemplation of poster with (*n* = 8) or without (*n* = 8) hypnosis, or no intervention (*n* = 10)	Dental surgery (USA)
Maranda 2015 [86]	Pilot Randomised Control Trial	Adolescents with type 1 diabetes mellitus	29	14.2 years	64%	Fishbowl containing *Betta splendens* fish, and instructions to pair diabetes self-management tasks with daily and weekly fish care duties, approximately 3 months	Usual care (*n* = 12)	At home (USA)
Riddick 1985 [60]	Between-subjects study without randomisation	Older adults in publicly subsidised housing	24	Range 57–94 years	71%	2.5-gallon tanks containing two goldfish, plus nine visits from researcher over six-months (*n* = 7)	Ten visits from the researcher over six-months (*n* = 8), or no intervention (*n* = 7)	Publicly subsidised housing (USA)
Sanchez 2015 [63]	Before-and-after study with control group	Students/trainees in paediatric orthopaedics	69	28.2 years	58%	30-minutes viewing 265-gallon saltwater aquarium with >25 fish, including several species of surgeonfish (*n* = 69)	30-minutes viewing white wall (*n* = 12)	Hospital waiting room (France)
Wells 2005 [68]	Between-subjects study with randomisation	University students	100	19.7 years	42%	10-minute video of 10 neon tetras swimming in a tank (*n* = 20)	10-minute video of birds in an aviary, primates in a zoo enclosure, a popular soap opera, or a blank screen (all *n* = 20)	University laboratory (UK)
*Public aquariums*								
Cracknell 2016 [87]	Between-subjects study, quasi-experimental	University students	84	24 years	76%	10-minutes viewing above exhibit when fully stocked (*n* = 29) or partially stocked (*n* = 26)	10-minutes viewing exhibit when unstocked (*n* = 29)	Public aquarium exhibit (UK)
Cracknell 2017, Study 1 [61]	Within-subjects study	University students	39	19.5 years	74%	Images of public aquarium exhibits (*n* = 39)	Images of built, green, aquatic or sub-aquatic environments (all *n* = 39)	University laboratory (UK)
Cracknell 2017, Study 2 [61]	Within-subjects study	University students	40	20.8 years	68%	Images of public aquarium exhibits differing in species richness, abundance of individuals, and content (tropical/temperate) (all *n* = 40)	-	University laboratory (UK)
Sahrmann 2016 [88]	Before-and-after study	General public	165	Range 18–68 years	72%	10-minute interaction with stingrays at touch-tank (*n* = 165)	-	Public aquarium exhibit (USA)

NR: not reported

**Table 3:** Summary of key findings of included studies.

First author and year	Procedure	Psychological outcomes	Physiological outcomes	Risk of bias*
*Fish as companion animals*				
Kidd 1999 [81]	Customers of shop selling fish and aquarium equipment completed survey.	*Benefits of aquarium ownership*: reported by 94% of respondents and included relaxation (*n* = 47), watching movements (*n* = 32), stress reduction (*n =* 23), companionship (*n* = 5), entertainment (*n* = 4) and education (*n* = 1).	-	-
Langfield 2009 [52]	Semi-structured interviews conducted with current pet fish owners; the data were analysed using the constant comparison method.	Four themes identified: *reasons for owning fish as pets; the environment; caring for pet fish;* and *benefits and limitations of owning fish as pets*.	-	+
*Correlational studies*				
Lin 2013 [82]	Medical directors from all accredited hospitals in Taiwan were mailed a questionnaire, which assessed patient-related work stress, the presence of five interior amenities in the workplace (including aquariums), and self-rated health status.	*Self-rated health (compared to same age population*, *compared to medical peers*, *short-term health complaints*, *long-term health complaints)*: no relationship between presence of an aquarium in the workplace, and any dimension of self-rated health was found.	*Self-rated health (short-term health complaints*, *long-term health complaints)*: no relationship between presence of an aquarium in the workplace, and any dimension of self-rated health was found.	+
*Intervention studies*				
Barker 2003 [53]	Patients assigned to rooms with/without aquarium on subsequent visits. Physiological outcomes assessed immediately after assignment and before treatment, psychological outcomes assessed before treatment only.	*Anxiety*, *depression*, *fear & frustration (VAS)*: no significant differences between aquarium and no aquarium conditions; trend towards a greater reduction in anxiety in the aquarium condition (*p* = 0.08).	*HR/DBP/SBP*: no significant differences found between aquarium and no aquarium condition before or after treatment.	+
Buttelmann 2014 [62]	Participants about to give spontaneous presentation interacted with fish/dog/plant/nothing for 5-minutes. Outcomes assessed at baseline, following induction of anxiety (being informed of the presentation task) and after the interaction.	*Anxiety (STAI-S)*: reduced significantly more in fish, dog and plant groups than no activity group; no significant differences between experimental groups. More participants in the dog group experienced a reduction to below baseline levels than those in control group; no differences between other groups. *Laughter (yes/no)*: more participants in the dog group laughed during the intervention that in all other groups; no differences between the other groups.	*DBP/SBP*: NR as influenced by participants’ movement/speech.	-
Cole 2000 [83]	Aquariums installed into the hospital rooms of patients awaiting heart transplantation. Outcomes assessed at baseline then after 3 and 11 days.	*Anxiety*, *depression*, *hostility*, *dysphoria*, *sensation seeking & positive affect (MAACL-R)*: no significant differences between baseline and follow-up.	-	-
DeSchriver 1990 [84]	Participants seated comfortably and watched live fish/fish video/placebo video for eight minutes. An emotive article was read aloud to induce stress. Outcomes assessed every minute during procedure.	*Treatment evaluation (adapted LSS)*: all conditions were perceived as equally relaxing.	*HR*, *skin temperature (°F)*, *muscle tension (μV)*: no significant differences between conditions.	-
Edwards 2002 [64]	Aquariums/picture installed into dining rooms of specialised dementia units. Nutritional intake assessed daily for two weeks before and after installation, then weekly for six weeks. Body mass assessed at baseline then monthly for four months.	-	*Nutritional intake (grams consumed/meal)*: significantly increased in two weeks after installation, then again in following six weeks. No significant changes in control group two weeks after installation. *Body mass (lbs)*: significantly increased in month after installation, then continued to increase until end of study (total *M* gain = 1.65lb).	-
Edwards 2013 [65]	Aquariums installed into dining rooms. Nutritional intake assessed daily for two weeks before and after installation, then weekly for six weeks. Average body mass was calculated for three months prior to installation (baseline), the intervention period (weeks 3–5), and follow-up (week 10 to 3-months post-intervention).	-	*Nutritional intake (grams consumed/meal)*: significantly increased in the two weeks after installation, but not in following six weeks. *Body mass (lbs)*: significantly increased from baseline to intervention but not from intervention to follow-up (total *M* gain = 2.2lbs).	-
Edwards 2014 [54]	Aquariums installed into dining rooms and outcomes assessed at baseline and after 10 weeks.	*BPSD (Nursing Home Disruptive Behaviour Scale)*: significant improvements on domains of uncooperative, irrational, sleep and inappropriate behaviours but not annoying or dangerous behaviours. Significant overall improvement. *Job satisfaction (Assessment of Work Environment Scale)*: significantly improved following introduction of the aquarium.	-	-
Katcher 1984 [85]	Participants received intervention immediately prior to treatment. Physiological measures assessed throughout intervention/procedure; psychological measures assessed during/after procedure.	*Treatment comfort (Treatment Comfort Index)*: patients rated comfort as significantly higher after aquarium contemplation than poster contemplation. Also higher in both aquarium groups and the poster with hypnosis group than the no contemplation group. *Anxiety (assessed by blind observer using a checklist) & patient compliance (assessed by dentist)*: no significant effect of aquarium observed.	*DBP/SBP*: no significant differences during intervention or procedure. *HR*: NR.	-
Maranda 2015 [86]	Participants obtained pet fish and were instructed to pair twice daily feeds with blood glucose readings, and weekly water changes with a parental review of their glucose logs. Outcomes assessed at baseline and follow-up (approximately 3 months).	*Quality of life (PedsQoL Generic and Diabetes modules)*: no significant effects were found for generic or health-related quality of life.	*Glycaemic control (A1C)*: significant reduction in A1C level for those in the intervention group compared to those in the control group. Younger participants (10–13 years) had a significantly greater response to the intervention than older participants (14–17 years).	+
Riddick 1985 [60]	Aquarium/visitor interventions were provided over six months. Outcomes were assessed via interview at baseline and six months.	*Leisure satisfaction (LSS)*: no significant difference between groups; one component (relaxation) bordered on significance (*p* = 0.06). *Loneliness (UCLA Loneliness Scale)*, *happiness (MUNSH)*, *anxiety (STAI-T)*: no significant differences between groups.	*DBP*: analysed as change from baseline to six-months for each group separately, due to differences at baseline. Only aquarium group underwent significant reduction. *SBP*: no significant differences between conditions.	-
Sanchez 2015 [63]	Participants watched aquarium continuously for 30-minutes. Outcomes assessed at 5, 10, 20 and 30-minutes, then at 10-minutes post-viewing.	-	*Pain threshold (measured using electrical stimulation device)*: significantly higher 5, 10, 20, and 30-minutes after viewing, compared to the initial values and remained elevated 10-minutes after viewing ended. No significant changes in the control condition.	-
Wells 2005 [68]	Participants watched one of the videos for 10-minutes then completed a reading aloud task designed to induce stress. Outcomes assessed at baseline (phase 1), after watching video (phase 2), and after reading task (phase 3).	-	*HR/SBP*: significantly lower in phase 3 in animal video groups compared to control video groups. No difference between animal videos groups. *DBP*: significantly lower in phases 2 & 3 in animal video groups compared to control video groups. No difference between animal videos groups.	+
*Public aquariums*				
Cracknell 2016 [87]	Participants viewed aquarium exhibit for 10-minutes; outcomes were assessed at baseline, 5-minutes and 10-minutes.	*Valence (Feeling Scale)*: a significant effect of time showed that valence increased with viewing; there was no significant effect of stocking level. *Arousal (Felt Arousal Scale)*: a significant effect of time showed that arousal significantly decreased with viewing; there was no significant effect of stocking level.	*DBP/SBP*: no significant differences were found between different stocking levels. *HR*: a significant effect of stocking level indicated that participants in the two stocked conditions had greater reductions in HR than those in unstocked condition.	-
Cracknell 2017, Study 1 [61]	Participants viewed each image and rated them on four dimensions.	*Attractiveness*, *willingness to display & affect*: built environments rated lower than all others, aquatic environments and aquariums rated highest. *Perceived restorativeness*: built environments rated lower than all others, aquariums rated higher than sub-aquatic and green environments, aquatic environments rated higher than aquariums.	-	+
Cracknell 2017, Study 2 [61]	As above.	*Attractiveness*, *willingness to display*, *affect & perceived restorativeness*: vertebrates rated higher than invertebrates; tropical exhibits rated more highly than temperate exhibits; high abundance rated higher than low abundance; high species richness rated higher than low species richness in tropical scenes but lower in temperate scenes.	-	+
Sahrmann 2016 [88]	Participants interacted with stingrays at the touch-tank. Physiological outcomes assessed throughout, psychological outcomes assessed pre- and post-interaction.	*Hedonic tone*: significantly improved from pre- to post-touch. *Energetic arousal*: significantly increased from pre- to post-touch. *Tense arousal*: significantly decreased from pre- to post-touch. (All assessed using the UMACL)	*HR*: significant quadratic trends showed that HR became more elevated and less variable during touch, then began to return to normal towards the end of the touch period (but did not reach baseline levels).	-

NR: not reported; BPSD: behavioural and psychological symptoms of dementia; DBP: diastolic blood pressure; HR: heart rate; LSS: Leisure Satisfaction Scale; MAACL-R: Multiple Affect Adjective Checklist-Revised; MUNSH: Memorial University of Newfoundland Scale of Happiness; SBP: systolic blood pressure; STAI-S: State-Trait Anxiety Inventory State Scale; STAI-T: State Trait Anxiety Inventory Trait Scale; UMACL; University of Wales Institute of Science and Technology Mood Adjective Checklist; VAS: visual analogue scales

*Risk of bias was assessed using the NICE (2012) Quality Appraisal Checklists for quantitative intervention studies and qualitative studies. ++ indicates all/most criteria were fulfilled and conclusions are unlikely to alter; + indicates some criteria were fulfilled and conclusions are unlikely to alter;—indicates few or no checklist criteria were fulfilled, and conclusions are likely or very likely to alter.

The broad inclusion criteria meant there was substantial clinical and methodological heterogeneity between included studies, and so statistical meta-analysis was deemed inappropriate. Therefore, a narrative synthesis of the evidence was conducted using techniques described by Popay et al. [89]. A preliminary synthesis was developed using textual descriptions, tabulation, groupings and clusters; the relationships within and between studies were then explored through qualitative case descriptions and the use of idea webbing/conceptual mapping. The robustness of the synthesis was assessed by reflecting on the methods used in the review, and by considering the quality of included studies and the strength of the evidence. Findings from studies conducted with existing home aquaria owners (*n* = 2), and those using correlational designs (*n* = 1), are discussed independently from those involving novel interactions with fish in aquariums (*n* = 16); as four studies in the latter group related specifically to public aquariums they are also considered separately.

### Fish as companion animals

Two studies were conducted with individuals who currently kept fish as companion animals to gain an understanding of their experiences. One was a phenomenological study which explored experiences of pet fish ownership through in-depth interviews (*n* = 9, *M* age = 34.9 years, 33% female) [52], while the second utilised a survey design and provided descriptive statistics on qualitative aspects of keeping home aquaria (*n* = 100, *M* age = 37.1 years, 50% female) [81]. Both studies identified relaxation and stress reduction as potential benefits of keeping fish as companion animals; this appeared to be primarily associated with watching the movements of the fish, although the sound of running water was also mentioned by some participants in one study [52]. Companionship was also identified as a potential benefit of keeping fish, although this was experienced to a much lesser extent than relaxation, with only a minority (5%) of participants in one study reporting this benefit. Other benefits associated with keeping fish included happiness [52], entertainment, and education [81]. A small number of participants (6%) in one study viewed their fish tanks as room decoration only [81]. Limitations to keeping fish were also identified. In one study, it was noted that fish cannot provide the same level of emotional support as other types of animal, and that participants had to deal with the death of their animals on a regular basis [52]. These factors were associated with variation in the level of attachment participants felt to their fish, with some reporting being highly attached, and others viewing their fish as replaceable [52]. Other limitations that were identified were associated with the maintenance, cost and time commitments of keeping home aquaria.

### Correlational studies

One study used a correlational design to assess whether the presence of aquariums in the workplaces of hospital medical directors was associated with their self-rated health. Participants (*n* = 737, mean age = 49 years, 8% female) were mailed a questionnaire to assess their patient-related work stress, and the presence of five interior amenities (aquariums, indoor plants, music, art and exhibitions, and private or personalised workspaces) within their working environment. Four dimensions of self-rated health were also assessed, specifically: how participants rated their own health against that of the same age population and their medical peers, and their experience of various health complaints during the past month (short-term) and six months (long-term). Both physiological and psychological health complaints were included. The analyses indicated that after controlling for personal characteristics, work status and work stresses, the presence of interior amenities was associated with an improvement in participants’ self-rated health. However, the presence of aquariums alone was not significantly related to any dimension of medical directors’ self-rated health.

### Intervention studies

Sixteen studies involved novel interactions with fish in aquariums, however, four of these related specifically to public aquariums so are discussed separately below. Of the twelve remaining studies, three involved student or trainee samples [62,63,68] and nine involved clinical populations, specifically: residents of specialised dementia units [54,64,65], dental patients [85], electroconvulsive therapy patients [53], hospitalised patients awaiting heart transplantation [83], older adults [60,84], and adolescents with type 1 diabetes mellitus [86]. One study also explored how staff were affected by the intervention [54]. Most studies involved adult populations, and where reported, mean ages ranged from 19.7 to 82.2 years, although student samples were typically younger than those drawn from clinical populations. One study involved adolescents and reported the mean age to be 14.2 years. All samples included male and female participants (20 to 92% female), with the exception of one study which did not report gender [85]. Ethnicity was reported in four studies; in three cases the sample was predominantly Caucasian (72.8 to 98.5%) [54,64,65]; the fourth consisted 56% Caucasian and 44% African American in the intervention group, and 33% Caucasian, 58% African American, and 9% other ethnicity in the control group [86]. Estimates of socio-economic status were reported in five studies; two reported level of education (65 to 68% high school educated or above) [54,64], one reported marital status (43% married) [53], one reported ZIP code-based annual household income (intervention group: $54,800; control group: $51,800) [86], and one reported that all participants qualified for “low income” publicly subsidised housing [60].

There was substantial variation in study setting and design. Two studies were conducted in university laboratories [62,68], one in a purpose-built laboratory in a housing complex [84], one in a hospital waiting room but under laboratory conditions [63], two in participants’ homes [60,86], and six in clinical or therapeutic settings [53,54,64,65,83,85]. Design of studies included before-and-after studies [54,83]; controlled before-and-after studies [63]; interrupted time series with [64] or without [65] control groups; within-subjects or crossover studies [53]; between-subjects studies with [68,84,85] or without [60,62] randomisation; and a pilot randomised control trial [86]. Where comparators were used they included no treatment or usual care controls, viewing alternative stimuli such as posters, and interacting with other animals (see Table 2 for further details).

The majority of studies used home aquaria containing between one and nine fish, with the exception of one study which used a 265-gallon aquarium with over 25 fish, installed in a hospital waiting room [63]. One study did not report details of the aquarium set-up [85], and while another study specified the species of fish which participants were to purchase (*Betta splendens*), it was not clear whether all participants adhered to these instructions [86]. Two studies used videos of fish in aquariums [68,84] and did not specify the size of the aquariums shown, although one was reported to contain ten neon tetras [68]. The type of interaction differed between studies and included: passive exposure [53,54,64,65]; actively watching the fish swimming [63,68,84,85]; interacting with a fish to “try and accustom it to humans” [62]; and caring for fish in an aquarium in either a hospital [83] or home [60,86] environment.

#### Anxiety & relaxation

Reflecting the findings of studies conducted with those who choose to keep home aquaria, several intervention studies (*n* = 7) assessed outcomes relating to anxiety or relaxation. A variety of instruments were used, and so meta-analytical techniques could not be applied. Two studies assessed whether brief exposure to an aquarium could alleviate anxiety associated with stress-provoking medical procedures. In Barker et al. [53], patients attending electroconvulsive therapy treatment were assigned to waiting rooms with or without aquariums. At the end of the waiting period (approximately 20 minutes) participants assessed their levels of anxiety using a visual analogue scale. Although participants reported lower levels of anxiety in the aquarium versus no aquarium condition, this did not reach a level of statistical significance. Katcher et al. [85] examined whether contemplation of an aquarium prior to dental surgery reduced anxiety during treatment; this was assessed by a blind observer who recorded overt signs of anxiety every five minutes throughout the procedure. Again, anxiety was lower in the aquarium groups compared to the comparator groups, but this difference was not statistically significant. However, scores from a Treatment Comfort Index indicated that patients who contemplated an aquarium before their dental surgery reported higher levels of comfort during the treatment than those who contemplated a poster or a blank wall. No differences were found in patient compliance as assessed by the dentist.

Buttelmann and Römpke [62] also assessed short-term changes in anxiety, this time in relation to a public speaking task. Student participants were asked to complete a short presentation on an unfamiliar topic with just five-minutes to prepare; a five-minute intervention period followed the preparation time during which participants interacted with a fish, dog, or plant, or were simply told to wait. Anxiety was assessed at baseline, after the stressor, and after the intervention period using the State scale of the State-Trait Anxiety Inventory (STAI); a score for each participant was calculated as the percentage of induced anxiety that was reduced following the intervention, where induced anxiety was calculated as the change from baseline to post-stressor. Participants who interacted with the fish had a greater reduction in induced anxiety than participants who received no intervention, and this reduction was equivalent to that experienced by participants who instead interacted with a dog or a plant. However, significantly more participants in the dog group experienced a reduction in anxiety to below baseline levels, relative to the control group; there were no significant differences between the other groups with regards to this outcome.

Two studies assessed anxiety over longer intervention periods. No change in anxiety was found for patients awaiting heart transplantation three- or 11-days after fish tanks were installed in their hospital rooms, as measured using the Multiple Affect Adjective Checklist-Revised (MAACL-R) [83]. Similarly, older adults who were given fish to care for in their own home experienced no greater reduction in anxiety (measured using the Trait scale of the STAI) after six-months, than those who received visits from the researcher, or no intervention [60]. In the latter study however, there was a borderline significant (*p* = 0.06) increase in relaxation (a component of the Leisure Satisfaction Scale) for residents in the aquarium group, compared to the comparator groups; there were no significant differences between groups for overall leisure satisfaction.

Outcomes relating to anxiety were assessed in two further studies. In DeSchriver and Riddick [84] participants viewed either a live fish aquarium, a video of fish swimming or a placebo video of television lines and static; participants’ responses to a treatment evaluation questionnaire (adapted from the Leisure Satisfaction Scale) indicated that all activities were perceived to be equally relaxing. Finally, Edwards et al. [54] found that installation of aquariums into specialised dementia units was associated with a significant improvement in carer ratings of residents’ behavioural and psychological symptoms, assessed using the Nursing Home Disruptive Behaviour Scale. This improvement in behaviour also coincided with an increase in job satisfaction among staff members, measured using the Assessment of Work Environment Scale.

#### Physiological stress

Six studies assessed indicators of physiological stress, although these outcomes were not reported in one study as the readings appeared to have been influenced by participants’ movement and speech [62]. Heart rate and/or blood pressure were the most commonly assessed outcomes, and were typically measured using automated devices (although in one case the device used was not stated [60]). However, the number and timing of assessments varied across studies; combined with the heterogeneity in study and intervention design, this variation made the data unsuitable for meta-analysis.

Two studies assessed whether the presence or contemplation of an aquarium could reduce heart rate and blood pressure among people undergoing medical procedures [53,85], with neither study finding a significant effect on either variable (one study did not report the findings related to heart rate [85]). DeSchriver and Riddick [84] assessed differences in heart rate, skin temperature and muscle tension between participants who viewed a live fish aquarium, a fish video, or a placebo video. Heart rate was measured as beats per minute using an automated device, skin temperature in degrees Fahrenheit using a temperature meter mounted on the participant’s finger, and muscle tension in microvolts using bicep electromyography (EMG); no significant differences were found between conditions for any of these variables. Wells [68] however, found that participants who watched an animal video (fish, bird or primate) had lower heart rate and blood pressure (systolic and diastolic) after a subsequent reading aloud task than participants who watched a control video (soap opera or blank screen). Diastolic blood pressure was also significantly lower immediately after viewing the video for participants in the animal video groups compared to the control groups; there were no differences between the animal video conditions. One study examined changes in blood pressure over longer periods and found that diastolic (but not systolic) blood pressure was significantly reduced after six months for participants who were given fish to keep in their own home, but not for those who received visits from the researchers, or had no intervention [60].

#### Affective state

Four studies assessed a range of outcomes relating to participants’ current mood or affective state. The presence of an aquarium in waiting rooms had no effect on fear, frustration or aggression (assessed using visual analogue scales) for participants awaiting ECT treatment [53]. Similarly, responses to the MAACL-R showed no change in depression, hostility, dysphoria, sensation seeking, or positive affect among patients awaiting heart transplantation three- or 11-days after aquariums were installed in their hospital rooms [83]. One study assessed whether happiness (assessed using the Memorial University of Newfoundland Scale of Happiness) increased after six months for older adults who were given fish to care for in their own home, but no significant differences were found between participants in the fish group and those who were visited by the researcher, or received no intervention [60]. In Buttelmann and Römpke [62], videotapes of the intervention period were coded for occurrence of laughter (yes/no); significantly more participants in the dog group were observed laughing compared to all other groups, with no significant differences between the fish, plant and control groups.

#### Loneliness

Riddick [60] used the UCLA Loneliness Scale to assess whether loneliness improved for participants who were given a fish versus those who received visits from the researcher, or had no intervention. No significant difference was found between the three groups after six-months, although a trend towards reduced loneliness was seen for those in the visitor group (*p* = 0.08).

#### Nutritional intake & body mass

As weight loss is a risk factor for people with dementia [90], two studies examined whether introducing fish aquariums into the dining rooms of specialised dementia units could influence residents’ nutritional intake, and subsequently improve their body mass [64,65]. Nutritional intake was measured as the amount of food (in grams) consumed at each meal during the intervention period, and in both studies, was found to significantly increase in the two weeks following installation. This increase also continued over the following six weeks, but only to a statistically significant level in one study [64]. In addition, body mass significantly increased during the months following installation of the aquarium, with average weight gains of 1.65lbs [64] and 2.2lbs [65] at four months post-installation. No changes in nutritional intake were observed after two weeks in a non-equivalent control group (*n* = 17) used in the earlier study [64].

#### Pain

One study involving healthy adults explored whether watching fish in an aquarium could increase participants’ pain threshold [63]. Participants (*n* = 69) were seated in front of the aquarium and gripped an electrical stimulation device between their fingers; the device increased in intensity until participants indicated that they could feel the sensation. The procedure was then repeated, and participants instead indicated when they first experienced pain. Assessments made throughout the 30-minute viewing period indicated that, although there were no changes in sensation threshold, participants’ pain thresholds were significantly increased after 5-, 10-, 20-, and 30-minutes compared to baseline readings. They also remained elevated 10-minutes after viewing ended. No changes in pain threshold were detected among a subset of participants who were retested while viewing a blank wall for the same amount of time (*n* = 12).

#### Glycaemic control

One study assessed whether pairing fish care duties with diabetes self-management tasks could lead to improved glycaemic control for adolescents with type 1 diabetes mellitus [86]. Participants randomly assigned to the intervention group were provided with a fishbowl and related equipment, and purchased a fish using a gift card provided by the researchers. They were then instructed to pair twice daily feeds (morning and evening) with blood glucose readings, and weekly water changes with a caregiver review of their glucose logs. Glycaemic control was assessed via A1C (an indicator of average blood glucose levels over the preceding three months) at baseline and follow-up (approximately three months). A significant improvement was found for the intervention group compared to the control group (usual care), with younger participants (10–13 years) having a greater response to the intervention than older participants (14–17 years). This study also assessed whether the intervention had an effect on generic and health-related quality of life, but no significant changes were observed from baseline to follow-up.

#### Secondary outcomes

Most intervention studies reported procedures that were in place to ensure animal welfare. In the three studies by Edwards and colleagues [54,64,65], the aquariums were specifically designed for use on a dementia unit; they were self-contained and locked to ensure the safety of fish and residents, and were automated to reduce the burden on staff. Two studies used aquarium servicing companies [53,83], with one also stating that participants were responsible for feeding the fish and had to place their initials on a feeding calendar to prevent under/over-feeding [83]. Buttelmann and Römpke [62] reported that when data collection was underway, one of five fish was taken from a communal tank and placed in a smaller goldfish bowl for the duration of the intervention, with the fish used in rotation to reduce stress; procedures were also in place to minimise stress experienced by the dog used as a comparator. Of the two studies in which participants kept fish in their own home, one reported that the tanks were initially maintained by the researchers and the participants, with the participants taking greater responsibility for fish care over the course of the study. Participants were also provided with information and literature on feeding and signs of fish illness, and could contact the researchers via telephone 24-hours a day [60]. The second study stated that participants were provided with instructions on how to care for their fish, but did not report any involvement in fish care on the part of the researchers [86]. This study also reported that the fish of two participants had to be replaced due to them dying during routine fish care, although it was not reported whether the cause of these deaths was known, or whether steps were taken to prevent future mortalities. Otherwise, no studies reported whether the welfare of the fish was affected due to their involvement in the research, so it is unclear whether these procedures were effective in practice. Similarly, no studies reported whether participants experienced adverse effects because of the interventions. Several studies provided anecdotal reports that participants responded positively to the interventions, however, none collected and reported these data in a rigorous and systematic manner.

### Public aquariums

Four studies reported in three papers related specifically to public aquariums. Two were conducted within an aquarium setting [87,88] and two (published in the same paper) used images of public aquarium exhibits [61]. Participants were either student samples [61,87] or healthy adults [88]. Sample sizes ranged from 39 to 165 (*M* = 82); all samples included male and female participants (68 to 76% female) and mean ages ranged from 19.5 to 24.0 years (one study reported only the age range of participants as 18 to 68 years [88]). Two studies were within-subjects designs with control groups [61], one was a before-and-after study [88], and one was between-subjects with pseudo-randomisation [87]. Interactions included very brief exposure to images [61], viewing a 550,000-litre exhibit at various levels of stocking [87], and physically interacting with stingrays at a touch-tank [88].

Two studies [87,88] examined changes in self-reported mood and physiological outcomes after interacting with fish at public aquarium exhibits. In Cracknell et al. [87], assessments using the Feeling Scale and Felt Arousal Scale indicated that participants’ affective state significantly improved, and their levels of arousal significantly reduced after viewing an aquarium exhibit for 10-minutes, with no significant differences between three levels of stocking (unstocked, partially stocked and fully stocked). Similarly, blood pressure reduced in all conditions but there were no significant differences between the stocking conditions. However, heart rate was influenced by level of stocking, with significantly greater reductions observed in the partially and fully stocked conditions compared to the unstocked condition. In Sahrmann et al. [88], heart rate was found to be elevated and less variable while interacting with stingrays at a touch-tank, compared to before or after contact with the animals. Three dimensions of mood were assessed using the University of Wales Institute of Science and Technology Mood Adjective Checklist (UMACL); hedonic tone and energetic arousal were significantly increased, and tense arousal was significantly decreased, after the 10-minute interaction period compared to before contact. This is indicative of a short-term increase in physiological stress when touching the animals, but a decrease in mental stress after leaving the exhibit.

In two studies [61], participants rated photographs of different environments on four dimensions of preference; the pleasantness of the scene, how willing they would be to display the image, how the image made them feel, and–of most relevance to this review–the perceived restorativeness of the scene. In study 1, public aquarium exhibits were compared to different natural and manmade environments. Aquariums were rated equally to, or higher than all other environments for all dimensions except perceived restorativeness, which was higher for aquatic environments (e.g. coastal landscapes). In study 2, different aquarium exhibits were compared. The findings indicated that tropical exhibits were preferred over temperate ones, and vertebrates were preferred over invertebrates; higher levels of biota were also preferred, but preference for species richness differed according to whether the exhibit was tropical or temperate (see Table 3 for further details).

#### Secondary outcomes

Of the studies relating to public aquariums two involved live animals; both used procedures which reflected typical visitor behaviours, and so animal welfare concerns were unlikely to be increased as a result of the research. Furthermore, Sahrmann et al. [88] reported that participants were shown proper touch techniques before being allowed to interact with the stingrays. Additionally, both studies asked participants about their feelings towards the exhibits. In Cracknell et al. [87] participants’ responses to evaluative statements indicated that they enjoyed watching the exhibit, found it interesting, felt better after watching, and would be happy to watch again. Furthermore, as the levels of stocking increased, participants’ responses became more positive. In Sahrmann et al. [88], 80% of participants gave positive responses to an open-ended question about their experience of the exhibit. Adverse events to participants were not reported in either study, however, Sahrmann et al. [88] stated that 7% of participants indicated they felt nervous, anxious or unsure when touching the animals. As the two studies by Cracknell et al. [61] used photographs in a laboratory setting, adverse events to participants were unlikely to have occurred, and there were no animal welfare concerns.

### Risk of bias in included studies

#### Qualitative studies

Two studies were assessed using the NICE Quality Appraisal Checklist for qualitative studies [78]. Of these two studies, one used methods appropriate to the study aims and took steps to ensure rigor and trustworthiness [52]. However, the use of snowball sampling and a lack of clarity regarding whether two researchers were involved in the entire analytical process introduced some risk of bias to this study. By contrast, the second study consistently lacked sufficient detail to make sound judgements about risk of bias and the defensibility of conclusions [81]. The study aims were not clearly stated, and there was insufficient description of the methods and data analyses to determine whether these were appropriate. As such, the findings of this study should be treated with caution. Further details are provided in S4 Appendix.

#### Quantitative studies reporting a correlation

One study [82] was assessed using the NICE Quality Appraisal Checklist for quantitative studies reporting correlation/association [78]. This study involved a survey design to assess whether the presence of interior amenities (including aquariums) is related to self-rated health among hospital medical directors; therefore, the research relied on self-report data which can be highly subjective. Additionally, while the study controlled for personal characteristics, work status and work stresses, it is unlikely that all possible confounders were considered; health-related behaviours and pre-existing medical conditions for instance, were not assessed but are likely to impact self-rated health status. Finally, the response rate was fairly low (32.83%), so it is unclear whether the included participants were representative of the source population.

#### Quantitative intervention studies

The remaining sixteen studies were assessed using the NICE Quality Appraisal Checklist for quantitative intervention studies [78]. One study was reported as a (pilot) randomised controlled trial (RCT)–usually considered the most rigorous form of experimental study design–and three others used between-subjects designs with random allocation [68,84,85]. Each of these studies had high or unclear risk of bias (see S4 Appendix for further details). One study reported that a randomisation schedule was developed using a computerised random number generator, and that this sequence was concealed until allocation, however, it was not clear how participants were allocated to this sequence [86]. No other studies reported the method of randomisation or whether allocation was concealed. One study reported that participants in the intervention and control groups were similar at baseline [86], and a second stated that groups were balanced in terms of age and gender, although participants’ baseline scores appeared to differ across conditions [84]. The remaining two studies did not report whether groups were similar at baseline [68,85].

Non-randomised, between-subjects designs were used in three studies [60,62,87]. One allocated participants on the basis of specific characteristics to ensure similarity between groups at baseline; whether balance was truly achieved is unclear however, as the authors did not provide descriptive statistics to support this statement [62]. Allocation could not be randomised in the second study due to the demands of the study site, and some differences between groups were observed at baseline [87]. In the final study, the control group was handpicked by the centre manager, and allocation to intervention conditions was based primarily on participants’ preferences; thus this study is subject to substantial bias [60].

Within-subjects or crossover designs were used in three studies, reported in two papers [53,61]. In two studies, the order of presentation was randomised and determined by a computer, thus concealing allocation [61]. In the third, participants were allocated on a first-come basis due to the needs of the service and allocation was not concealed [53].

Six studies used before-and-after [54,63,83,88] or time series [64,65] designs. Although two [63,64] included a small number of participants from the treatment group in a control group, both analysed results from each group separately and based conclusions predominantly on findings from the treatment group in isolation, therefore, these two studies are considered alongside those without a comparator. Uncontrolled designs are typically considered to have low internal validity, and as only two studies interpreted results in relation to existing trends [64,65], it is unclear whether the results observed in these studies differ from those which would have been observed naturally over time.

Several potential sources of bias were present across included studies, irrespective of design. Blinding of participants was impossible due to the nature of the interventions (as people can see the fish), although some studies did report that participants were unaware of the study aims during data collection [53,68,87]. Three studies reported blinding of study personnel [53,62,85] but in two cases this applied only to certain aspects of data collection, specifically, scoring of visual analogues scales [53] and coding of videos [62]. Only one within-subjects study reported using a sufficient washout period between conditions [64], and contamination may have been an issue in studies where participants had access to the fish tank between testing sessions [84], or could have visited neighbours assigned to the aquarium group [60]. Most studies provided an adequate description of the aquariums, although additional details such as the species of fish used would have been beneficial in some cases; only one study provided no details of the aquarium set-up [85]. In two studies, adequacy of exposure could be questioned; Sahrmann et al. [88] reported that visits to the touch-tank typically last around 20 minutes, but the intervention period lasted only ten, while Cole and Gawlinski [83] stated that the type of patients included in their study usually experience a waiting time of two months, but the longest follow-up was just 11-days (although it is noteworthy that this was a pilot study). In one study, exposure to the intervention and control were non-equivalent by design of the research [64], and in another it was unclear if length of exposure was standardised, or determined by the participant [61].

In some studies, methods of statistical analysis were unclear or inadequately reported [53,60,62,83–86]. Several inappropriately used multiple comparisons in place of a single omnibus test [61,63–65,87] and some also used parametric tests for Likert-type data which may be considered inappropriate (although this is highly debated) [54,61,87]. Only two studies reported conducting power analyses. In one study these were conducted on a *post-hoc* basis and so are of limited value [53], while in the second the data used as a basis for the power analysis was not clearly described [86]. Dropout rates were reported in only two studies [54,60] and while both were at an acceptable level (<10%), neither conducted an intention-to-treat analysis. One study reported excluding a participant because they bought a fish when assigned to the control group [86]. The problem of missing data was discussed in a further four studies [53,62,87,88] and was typically dealt with by excluding participants with incomplete data from the analyses.

Only two studies adequately described the recruitment and selection process [60,88], but one of these reported that participants for the control group were handpicked by the service manager (thus risk of bias was high) [60]. Most other studies provided some details such as the inclusion/exclusion criteria, but these were insufficient to determine whether risk of bias was minimised. Descriptions of participant characteristics were limited in most studies, with one providing no information about the sample [85]. For these reasons, it is unclear whether the findings from most studies are generalizable to the source populations.

### Reporting biases

Evidence of potential selective reporting was identified in some studies. Katcher et al. [85] reported collecting heart rate data but this outcome was not included in the results section of the article. Similarly, in discussion of their research findings, Edwards and Beck [64] referred to data which indicated reduced use of nutritional supplements among participants, but this outcome was not discussed in either the methods or results sections. A third study [62] did not present data collected regarding heart rate and blood pressure, however the authors explained the reason for this exclusion; the data failed to show successful anxiety induction in over 50% of participants, and may have been influenced by participants’ movements and speech. Aside from omission of specific outcomes, some studies did not sufficiently report results, for instance failing to provide full details of the statistical test and significance levels [83], or not reporting the results of *post hoc* testing [60]. For one study, a lack of detail regarding the methodology made it difficult to determine whether results were presented in full [81]. Due to heterogeneity in study outcomes it was not possible to assess for evidence of publication bias using statistical methods (i.e. funnel plots). However, given that included studies were limited to those published in the English language, and that additional research was identified during the search but could not be accessed, it is probable that some relevant research was unintentionally omitted.

### Strength of evidence

Strength of evidence assessments were made using the Weight of Evidence approach [80]. For this review, ratings of study quality (*Weight of Evidence A*) corresponded directly to the assessments made using the NICE Quality Appraisal Checklists [78] (see Table 3). Studies with high risk of bias (-) were rated as ‘low’ (*n* = 12) and studies with unclear risk of bias (+) were rated as ‘medium’ (*n* = 7). As no studies were assessed as having low risk of bias (++), no studies were rated as ‘high’ for this criterion (see Tables 4 and 5 for more details).

**Table 4:** Weight of evidence assessments for psychological outcomes.

First author and year	Weight of Evidence A: Quality	Weight of Evidence B: Research design	Weight of Evidence C: Relevance	Weight of Evidence D: Overall weight
*Fish as companion animals*				
Kidd 1999 [81]	Low	Low	Low	Low
Langfield 2009 [52]	Medium	Low	Medium	Medium
*Correlational studies*				
Lin 2013 [82]	Medium	Low	Low	Low
*Intervention studies*				
Barker 2003 [53]	Medium	Medium	High	Medium
Buttelmann 2014 [62]	Low	Medium	High	Medium
Cole 2000 [83]	Low	Medium	High	Medium
DeSchriver 1990 [84]	Low	High	High	High
Edwards 2014 [54]	Low	Medium	High	Medium
Katcher 1984 [85]	Low	High	High	High
Maranda 2015 [86]	Medium	High	High	High
Riddick 1985 [60]	Low	Low	High	Low
*Public aquariums*				
Cracknell 2016 [87]	Low	Medium	High	Medium
Cracknell 2017, Study 1 [61]	Medium	High	Medium	Medium
Cracknell 2017, Study 2 [61]	Medium	High	Medium	Medium
Sahrmann 2016 [88]	Low	Medium	High	Medium

**Table 5:** Weight of evidence assessments for physiological outcomes.

Study ID	Weight of Evidence A: Quality	Weight of Evidence B: Research design	Weight of Evidence C: Relevance	Weight of Evidence D: Overall weight
*Correlational studies*				
Lin 2013 [82]	Medium	Low	Low	Low
*Intervention studies*				
Barker 2003 [53]	Medium	Medium	High	Medium
DeSchriver 1990 [84]	Low	High	High	High
Edwards 2002 [64]	Low	Medium	High	Medium
Edwards 2013 [65]	Low	Medium	High	Medium
Katcher 1984 [85]	Low	High	High	High
Maranda 2015 [86]	Medium	High	High	High
Riddick 1985 [60]	Low	Low	High	Low
Sanchez 2015 [63]	Low	Medium	High	Medium
Wells 2005 [68]	Medium	High	High	High
*Public aquariums*				
Cracknell 2016 [87]	Low	Medium	High	Medium
Sahrmann 2016 [88]	Low	Medium	High	Medium

With regards to study design (*Weight of Evidence B*), studies were rated as ‘high’ if they used randomised experimental designs (*n* = 6), and ‘medium’ if they used other experimental designs (*n* = 9). The exception was the study by Riddick [60] which was rated as ‘low’ despite using a non-randomised experimental design; this was due to the inappropriate methods of allocation (participant choice and hand selection by the centre manager). Studies using qualitative or correlational designs (*n* = 3) were rated as ‘low’ because these studies did not directly assess how interacting with fish in aquariums influences well-being outcomes.

The majority (*n* = 14) of studies received a rating of ‘high’ for relevance (*Weight of Evidence C*), as the findings related directly to the review question(s). Two studies were downgraded to ‘medium’ as they used photographs and assessed the perceived restorativeness of various public aquarium exhibits, rather than measuring actual changes in restoration outcomes (e.g. mood, physiological stress) [61]. One qualitative study [52] was also downgraded to ‘medium’ as, while an insight into the psychological benefits of interacting with fish in aquariums was given, well-being outcomes were not directly assessed. The second qualitative study [81] was rated as ‘low’ because the data were extremely limited, and there was inadequate description of the aims, design and results to determine the relevance of the study. Finally, the one study using a correlational design [82] was given a rating of ‘low’ for relevance because aquaria were a very small part of the study, and no description was given regarding the nature of the aquariums or the ways in which participants interacted with the fish they contained.

The overall weight for each study (*Weight of Evidence D*) was assigned based on the most common rating from the first three criteria. Most studies (*n* = 12) achieved an overall weight of ‘medium’, with three rated as ‘low’ [60,81,82] and four as ‘high’ [68,84–86]. The lowest weighted evidence came from studies using correlational designs, or involving individuals who already kept fish as companion animals; two studies were rated as ‘low’ and one as ‘medium’. By comparison, evidence from the intervention studies was weighted more strongly; one study was rated as ‘low’, seven as ‘medium’ and four as ‘high’. Evidence from public aquarium studies was more consistent, with all four studies achieving an overall weight of ‘medium’.

With respect to the two review questions, psychological and physiological well-being were explored in fifteen and twelve studies, respectively. In both cases the majority of studies were assigned an overall weight of ‘medium’ (*n* = 9 for psychological outcomes, *n* = 6 for physiological outcomes). However, three studies relating to psychological well-being were rated as ‘low’ and three as ‘high’, whereas four studies assessing physiological well-being achieved an overall weight of ‘high’, with only two rated as ‘low’. This suggests that the evidence relating to the physiological benefits of interacting with fish in aquariums may be slightly stronger than that relating to the psychological benefits. It is noteworthy however, that many studies relating to both types of outcome were ‘upgraded’ due to the high relevance of the evidence and the suitability of research designs, but had a high risk of bias (see Tables 4 and 5 for details). Thus, while research related to physiological outcomes may be slightly stronger than that relating to psychological outcomes, overall the strength of evidence was fairly low and substantial limitations were present in both evidence bases.

## Discussion

The purpose of this review was to investigate the psychological and physiological benefits of interacting with fish in aquariums. Nineteen studies were included in the review, encompassing those relating to the benefits of keeping fish as companion animals, correlational studies, and research involving novel interactions with fish in home or public aquariums. The strength of evidence relating to physiological outcomes was slightly higher than for psychological outcomes, however both evidence bases had mixed results. Of the fifteen studies that explored psychological outcomes, four studies of medium weight observed positive effects associated with human-fish interactions [52,54,62,88], but six (*n* = 1, high; *n* = 3, medium; *n* = 2, low) found only partial support [60,61,81,85,87] and five (*n* = 2, high; *n* = 2, medium; *n* = 1, low) found no effect [53,83,84,86]. Similarly, a positive effect on physiological outcomes was observed in six of twelve studies (*n* = 2, high; *n* = 4, medium) [63–65,68,86,88], but partial support was found in two (*n* = 1, medium; *n* = 1, low) [60,87], and no effect in four (*n* = 2, high; *n* = 2, low) [53,84,85]. Furthermore, there was substantial clinical and methodological heterogeneity between included studies, and risk of bias was either high or unclear. These factors must therefore, be considered when drawing conclusions from this collection of studies.

Reflecting the widespread belief that watching fish swimming is relaxing, there was tentative evidence from two studies (rated ‘low’ and ‘medium’ for weight of evidence) that keeping fish as companion animals is associated with benefits such as stress reduction and increased relaxation. Other benefits were also identified, including happiness and companionship. Not all participants shared these experiences however, with some reporting no benefits from keeping home aquaria. This reflects the inconsistency of research findings more widely in the field of HAI, specifically, the lack of conclusive evidence to demonstrate a direct link between companion animal guardianship (irrespective of the type of animal) and improved well-being [21].

One possible explanation for these inconsistent findings is that the benefits of companion animal guardianship are mediated by the strength of the attachment between a human and their companion animal [41,91,92]. In both studies, variation was apparent in the level of attachment fish owners had to their animals; some participants indicated a strong attachment bond, while others indicated difficultly in forming attachments due to a lack of reciprocal affection from their fish. This variation may therefore account for the divergent experiences of those who keep fish as companion animals. Similarly, there were differences between participants in the degree to which they were involved in the care of their fish. One study noted that most participants were responsible for feeding the fish themselves, but some shared this responsibility with other family members [81]; participants in the second study noted that different species require different levels of care [52]. As increased effort can lead to people placing greater value on the product of that effort [93], home aquaria owners who take a greater role in the care of their fish may perceive more benefits from their animals than those who take a lesser role. Alternatively, as research has indicated that the beneficial effects of companion animal guardianship may be mediated by factors such as sociodemographic characteristics and health-related behaviours [19,22,23], individual differences between people who keep fish may explain some of the variation in findings. Future research should therefore, consider how differences in attachment, involvement in companion animal care, and population characteristics may account for variation in the experiences of those who keep fish as companion animals.

The findings from intervention studies were largely inconclusive. In contrast to research with other animals such as dogs and cats (for meta-analysis see Ein et al. [94]), there was little evidence that fish aquarium-based interventions help to reduce psychological and physiological stress or anxiety. Promisingly, one highly weighted and two medium weighted studies found significant positive effects on outcomes related to physiological stress and anxiety or related outcomes [54,62,68]. However, two studies (one weighted high, one low) found only partial support [60,85] and three studies (one high, two medium) found no significant effects [53,83,84]. Similarly, while previous research has linked contact with animals to reduced loneliness [5,95], acquisition of a fish tank had no effect this outcome among older adults after six months (although the only study to explore this outcome was weighted low for strength of evidence, and had substantial risk of bias) [60]. As is common with research into HAI [25,26] however, many studies had small sample sizes and thus may have been underpowered. As several studies with smaller samples observed greater improvements in the aquarium versus comparator groups, the possibility that these differences may have become statistically significant given sufficient power cannot be rejected.

Alternatively, individual differences in participant characteristics may account for some of the variation in study findings. Ein et al. [94] for example, observed that pet therapy (with dogs and cats) reduced heart rate for healthy adults but not clinical samples and reduced subjective stress/anxiety among adults but not older adults. It was argued that these moderating effects may be due to the use of medication among clinical samples, and greater emotional stability in older adults. Although substantial clinical and methodological heterogeneity precluded the use of moderator and subgroup analyses in the current review, it is plausible that participant characteristics may have had similar moderating influences on the effectiveness of human-fish interaction. For instance, of the five studies assessing physiological stress, four involved clinical populations or older adults, and found either no effect [53,84,85] or only mixed evidence [60]. Conversely, the final study involved a student sample and found significant improvements in heart rate and blood pressure associated with watching videos of fish [68]. While medication usage was not considered within these studies, it is possible this discrepancy in results may be at least partially accounted for by a higher level of medication use among the clinical and older adult samples, compared to student samples.

There was some support for the effectiveness of fish aquarium-based interventions on pain [63], nutritional intake and body mass [64,65], but attribution of these effects to human-fish interaction is limited by poor study design. For instance, in the two studies by Edwards and Beck [64,65], staff members were required to track residents’ food intake by physically weighing their food at mealtimes; as nursing home staff often overestimate food intake among residents [96], tracking levels of consumption in this manner may have led to increases in residents’ nutritional intake simply by raising staff members’ awareness of undereating. As these (and other) studies lacked appropriate control groups, it is not possible to separate any effects of the aquarium from other factors associated with the research, or from any changes that would have occurred naturally over time [26]. Other common methodological concerns were the lack of randomisation, allocation concealment, and blinding of study personnel; it is noteworthy however, that these methodological concerns are not uncommon in other areas of HAI research [25,26].

In some intervention studies the observed benefits may have been attributable to factors other than human-fish interaction. For instance, one study found that pairing diabetes self-management tasks with routine fish care activities led to improved glycaemic control among adolescents with type 1 diabetes [86]. Arguably however, the fish may not have been an integral component of this intervention; self-management behaviours may be paired with any regular activity, such as mealtimes, and waking or sleep routines [97]. While it is possible that adolescents may have greater motivation to adhere to an intervention because it involves interaction with live animals [33], without direct comparison of fish care tasks to other routine activities, it is impossible to determine whether the fish were a necessary component of this intervention. Likewise, while Buttelmann and Römpke [62] found that interacting with a single fish in a goldfish bowl reduced anxiety to a greater extent than no intervention, this effect was equivalent for participants who instead interacted with a dog or a plant. Although a substantial body of research would predict benefit from interacting with a dog, it is less clear that these benefits should be observed from interacting with a single plant (although there is some evidence that the presence of indoor plants has psychological benefits (for overview see Bringslimark et al. [98]), this research usually focuses on passive exposure and so the interaction within the current study–brushing the leaves of the plant with water–was not typical). As such, the authors acknowledged that these findings may be attributed to simple distraction, and so could be achieved with a variety of other activities provided they are engaging for the individual [33,42].

Similarly, while the findings related to public aquariums were generally quite positive, it was not always clear whether these benefits were the direct effect of human-fish interaction. Cracknell et al. [87] observed improvements in all participants irrespective of whether any fish species were present in the public aquarium exhibit. This suggests that benefits may be experienced from exposure to underwater scenes even in the absence of live animals, a finding which better aligns with theories of restorative environments than HAI. Interestingly however, heart rate did improve to a greater extent as the abundance of fish increased, and higher stocking levels were associated with longer viewing times in a separate sample of participants [87]. Furthermore, photographs of aquariums were rated more highly when displaying a higher abundance of fish, although preference for diversity of species differed between tropical and temperate exhibits [61]. These findings suggest that there may be additional benefits to interacting with live animals compared to other stimuli; as there are risks and animal welfare concerns associated with HAI [12,51], additional research is needed to corroborate these findings.

### Strengths

The major strength of this review is that it is the first attempt to systematically examine the psychological and physiological benefits of interacting with fish in aquariums. One previous review has addressed this topic [76], but the narrative account focused on research from a restorative environments perspective and did not use a systematic approach. Consequently, evidence relevant to the current research questions was excluded, whereas the systematic approach used in the current review led to a more comprehensive overview of the research findings. The specification of inclusion/exclusion criteria also ensured that research met a minimum standard for inclusion; HAI research has often relied on evidence from poor quality sources that lack stringent peer review, such as books and conference proceedings [99], but evidence from these sources was excluded from the current review. While studies of any design were included, assessing risk of bias and the strength of the evidence ensured that the research findings were considered in the context of methodological limitations. Overall, by using a systematic approach and adhering to PRISMA guidelines [77], this review provided a more rigorous and reliable synthesis of the research evidence, while aiming to meet the standards of transparency and reporting widely expected in other areas of health-related research.

### Limitations

There are a number of limitations to this review which should be acknowledged. Firstly, bias may have been introduced by limiting included studies to those published in the English language. Furthermore, three potentially relevant studies could not be included due to issues in gaining access to the full-text, or for copyright reasons (in all cases attempts to identify/contact the authors were unsuccessful). As two of these studies were unpublished theses, this may signify the presence of publication bias; however, it was not appropriate to assess for publication biases statistically due to heterogeneity in study outcomes.

The major limitations of this review were however, the scope and quality of the current research evidence. While the inclusion criteria were deliberately broad to maximise results, only nineteen studies were included in the review; however, despite this small number of included studies, there was substantial clinical and methodological heterogeneity, which made it difficult to draw comparisons across research findings. Furthermore, in all studies risk of bias was either high or unclear, and the strength of evidence was fairly low, with only four of nineteen studies achieving a rating of ‘high’ for weight of evidence. While the identification of these limitations is crucial to support the development of future research, these inconclusive findings are unhelpful to practitioners wishing to provide their clients with evidence-based advice or interventions.

### Future directions

Reflecting the field of HAI more broadly, there is a need for future research to address the discussed methodological limitations, and minimise sources of bias. Intervention studies should aim to meet the “gold standard” of RCTs, or use appropriate alternatives where this is not a possibility (for overview see Kazdin [26]). Given the particularly low strength of evidence relating to keeping fish as companion animals, there is also a need for large-scale observational research to better explore the effects of home aquaria ownership on well-being; this could be achieved through the incorporation of questions about companion animal guardianship into existing longitudinal studies [19,28]. Both experimental and observational research should take into consideration any mediating effects of attachment, sociodemographic characteristics, and health-related behaviours. Furthermore, as examining commonalities in qualitative research findings may be key in identifying the mechanisms underlying HAI [26], there is a need for additional qualitative research on the topic of human-fish interaction.

Aside from addressing methodological limitations, there are several opportunities for future research highlighted by this review. While most of the intervention studies were conducted within specific clinical populations, there was preliminary evidence that human-fish interaction may be beneficial among non-clinical samples. For example, one study found that student anxiety decreased following brief interactions with a single fish in an aquarium [62], and another observed an improvement in job satisfaction among staff working in dementia units following the installation of fish aquariums (although it is unclear whether this was due to the presence of the aquariums or improvements in residents’ behaviour) [54]. These findings reflect research with dogs which has indicated that HAI may be beneficial in educational [100,101] or workplace environments [102]. Future research may therefore, wish to explore the influence of interaction with fish in aquariums on student or employee well-being, although it is noteworthy that one study found no relationship between hospital medical directors self-rated health and the presence of aquariums in their working environment [82]. Additionally, as all but one of the included studies were conducted within adult populations, it would be of interest to further explore whether interacting with fish in aquariums is beneficial for the well-being of child or adolescent participants.

Another potential avenue of investigation is to explore which aspects of fish aquariums contribute to improved well-being. Research in public aquariums indicated that the abundance of fish and diversity of species may have a positive impact on well-being outcomes [61,87], but it is currently unclear whether this translates into home aquaria. Furthermore, as this research observed benefits associated with exposure to an unstocked aquarium exhibit [87], future research should take into consideration the presence of additional aquarium features, such as other animals (e.g. snails, shrimp, corals), plants or ornaments, and whether the sound of running water produced by a fish tank plays a role in relaxation [52]. Similarly, consideration should also be given to the type of human-fish interaction. For instance, one study found positive effects associated with interacting with stingrays at an aquarium touch tank [88], but this interaction is very atypical in the context of this review. Thus, while the study furthers the evidence base regarding the benefits of human-fish interaction, it is unclear whether these effects will translate to the benefits of fish aquaria more broadly. Moreover, variation also exists within more common forms of interaction; being involved in the care of the animals may for example, lead to different effects than simply watching fish swimming, and the effectiveness of interventions may be influenced by intensity of exposure, such as the duration and frequency of the human-fish interaction.

Finally, future research should consider the impact of human-fish interactions on the animals involved. At present, much research into the health benefits of HAI has focused on human well-being, an approach which has been criticised as being human-centred [103,104]. Some researchers have therefore argued for a greater emphasis on the reciprocal nature of HAI, with animals considered active participants in human-animal encounters [20,104]. While research with other species (typically dogs) has begun to investigate the impact of HAI on the animals involved, the effects of human-fish interaction on the fish involved were largely absent from the studies in this review. One paper reported that the fish of two participants died during routine fish care and were replaced [86], but did not specify whether the cause of these deaths was known, or whether steps had been taken to prevent future mortalities. No other studies reported whether the fish experienced any adverse effects of the interactions, and no studies directly assessed fish welfare. Therefore, future research exploring the health benefits of interacting with fish in aquariums should at minimum report whether (or not) any adverse effects to animal welfare are experienced as a result of human-fish interactions.

Parallel to this, additional research is needed to determine the effectivity of non-live alternatives, such as videos of fish swimming. As such interactions provide exposure to animals (albeit in simulated form) while eliminating animal welfare concerns, they may provide a suitable substitution for live fish aquariums. At present only two studies (to our knowledge) have investigated the benefits of watching fish videos [68,84], with conflicting findings. More broadly however, research has identified that robotic animals may have positive effects on well-being outcomes, such as loneliness, depression, and anxiety in older adults [105,106]. Thus, it is possible that non-live alternatives to fish aquaria, such as videos, robotic fish, or computer simulations, may benefit human well-being while eliminating risks to both the human and the animal. However, more research is needed before conclusions can be drawn.

### Conclusion

The findings of this review provide tentative support that interacting with fish in aquariums may be beneficial for psychological and physiological well-being among humans. Although findings were mixed, many studies had small sample sizes, so it is possible significant effects would have been detected given adequate power. Conversely however, many studies were subject to methodological limitations and had high or unclear risk of bias. Therefore, more research is needed before firm conclusions can be drawn. Future research on this topic should be well powered, and aim to use robust methodologies that minimise potential sources of bias. Consideration should also be given to any factors which may influence the effects of human-fish interaction, such as participant characteristics, features of the aquarium, or the type of interaction between the human and the animals. Finally, the details of the study design, and in particular the human-fish interaction, should be clearly described to allow for replication.

## Supporting information

S1 Appendix PRISMA checklist. (PDF)

S2 Appendix Protocol. (PDF)

S3 Appendix Data extraction form. (PDF)

S4 Appendix Quality appraisal. (PDF)
