# Supplementary material for: The effects of interacting with fish in aquariums on human health and well-being: A systematic review
Source: PLoS One. 2019 Jul 29;14(7):e0220524. doi: 10.1371/journal.pone.0220524 (PMC6663029; doi:10.1371/journal.pone.0220524)
Supplement: S2 Appendix — (PDF) [file pone.0220524.s002.pdf]

PROSPERO
International prospective register of systematic reviews

National Institute for Health Research

The health and well-being benefits of interacting with fish in aquariums: a systematic review

Heather Clements, Stephanie Valentin, Nick Jenkins, Jean Rankin, Julien Baker, Donna Snellgrove, Nancy Gee, Katherine Sloman

Citation

Heather Clements, Stephanie Valentin, Nick Jenkins, Jean Rankin, Julien Baker, Donna Snellgrove, Nancy Gee, Katherine Sloman. The health and well-being benefits of interacting with fish in aquariums: a systematic review. PROSPERO 2018 CRD42018090466 Available from: http://www.crd.york.ac.uk/PROSPERO/display_record.php?ID=CRD42018090466

Review question

What influence does interaction with fish in aquariums have on the psychological well-being of human participants?

What influence does interaction with fish in aquariums have on the physiological well-being of human participants?

Searches

Searches will be conducted in the following electronic databases: CINAHL, Education Source, ERIC, Health Source – Nursing/Academic Edition, MEDLINE, PsycARTICLES, Psychology and Behavioural Sciences Collection, PubMed, SAGE Journals ONLINE, ScienceDirect, and Web of Science. Databases will be searched from inception until the date of searches (January 2018). This will be supplemented by searches in Google Scholar and on the following websites specialising in human-animal interaction: WALTHAM Science (www.waltham.com), HABRI-Central (www.habricentral.org), and Animals and Society Institute (www.animalsandsociety.org). The references sections of full-text articles and any relevant review articles identified during the searches will be hand searched for additional primary research.

Types of study to be included

Included studies will be limited to primary research, but there will be no further limitations on the study design. Both quantitative and qualitative research will be included. Research will be limited to that published in peer reviewed journals and doctoral theses.

Condition or domain being studied

Psychological and physiological well-being outcomes.

Participants/population

There will be no limitations on the participants of included studies.

Intervention(s), exposure(s)

Any form of interaction with fish in aquariums will be included, including animal-assisted interventions, lab-based experiments and studies of pet ownership. This will include passive exposure to an aquarium, actively watching fish in an aquarium, and caring for fish in an aquarium. "Non-live" alternatives (e.g. videos of fish swimming displayed on a screen) will also be considered. There will be no limitations on the length, frequency or duration of exposures. Research relating to the health benefits of fish consumption,

studies on well-being benefits of fishing/angling, and any studies involving invasive research conducted on fish will be excluded.

Comparator(s)/control

Studies both with and without a control group will be included. For those with a control group, any type of comparator will be considered, including: no treatment controls, alternative animal-assisted interventions, and alternative interventions without animal involvement.

Context

There will be no limitations on the setting of the research: clinical and laboratory based research will be included. Research relating to large scale public aquariums will also be included, but will be analysed separately from that relating to smaller home aquaria.

Primary outcome(s)

1. Psychological outcomes. Any psychological outcome relevant to well-being such as depression, anxiety, loneliness, health-related behavior change, social interaction. Where multiple measures are used, priority will be given to those measured with validated instruments (e.g. Beck Depression Inventory, State-Trait Anxiety Inventory).

2. Physiological outcomes. Any physiological outcomes relevant to well-being such as blood pressure, heart rate, weight change.

Secondary outcome(s)

1. Attitudes to fish/aquarium based interventions, or pet fish ownership.

2. Adverse effects on human health.

3. Issues regarding animal welfare.

Data extraction (selection and coding)

Study selection: All records identified via electronic databases will be exported into a single EndNote library and duplicates will be removed. All remaining records will then be screened in a two-stage process by two independent reviewers. Initially the title and abstract of all articles will be assessed for relevance, with any disagreements resolved through discussion or by a third reviewer. Next the full-text of all remaining articles will be obtained and screened against the inclusion/exclusion criteria; reasons for exclusion of full text articles will be recorded and any disagreements will be resolved through discussion or by a third reviewer. The reference lists of all included studies will then be screened for additional primary research and the human-animal interaction websites will be searched for additional research.

Data extraction: Data from included studies will be extracted using a purpose-developed data extraction form; this will be completed by two independent reviewers and any disagreements will be resolved through discussion with a third reviewer. The following information will be extracted: general details (author, year, publication type, county, funding and conflicts of interest); study details (aims/objectives, dates of data collection, theoretical framework); methods (study design, recruitment, participant characteristics, intervention, control condition, allocation, setting, primary outcomes (psychological outcomes, physiological outcomes), secondary outcomes (adverse events, animal welfare, attitudes to fish/aquarium based interventions), brief study description); results (analysis, primary outcomes, secondary outcomes); author conclusions; and reviewer comments.

Risk of bias (quality) assessment

Due to the broad inclusion criteria it is likely that included studies will incorporate both quantitative and qualitative research. Therefore, to reflect this variation in study design, quality will be assessed using the National Institute for Health and Care Excellence Quality Appraisal Checklists (2012); checklists are available for intervention studies, quantitative studies reporting a correlation or association, and qualitative studies. Quality assessment will be conducted by two independent reviewers; any disagreements will be resolved through discussion with a third reviewer.

Selective reporting of outcomes will be assessed by comparing the methods section of included studies to the presented results, to identify any discrepancies which occur and assess whether an adequate description of the results is provided. If there is reference made to a published or registered protocol, comparisons will also be drawn between the protocol and published results. Where possible, publication bias will be assessed through identification of asymmetry in funnel plots; however, as this method is not recommended for outcomes with fewer than ten studies it will therefore be utilized only where a sufficient number of studies are identified.

Strategy for data synthesis

Data will be analysed using a narrative synthesis, following the approach outlined by Popay et al. (2006). Initially textual descriptions and tabulation of study data will be used to develop a preliminary synthesis; this will be grouped according to the two primary outcome categories (psychological/physiological). Next relationships in the data will be identified by exploring subgroups and moderator variables. Finally, the robustness of the synthesis will be assessed by examining the quality of included studies (quality appraisals) and assessing the risk of bias (publication bias, selective reporting) across studies.

It is anticipated that there will be too great a level of clinical and methodological heterogeneity between studies for meta-analysis to be appropriate; this will be assessed by comparing participant characteristics, intervention characteristics, measured outcomes, and methodological design across included studies. However, where meta-analytical techniques are deemed appropriate, a random-effects model will be used. This model was chosen over a fixed-effect model as it is typically more conservative and accounts for variation in treatment effects. However, as random-effects models can be influenced by small studies, a fixed effect model will also be used where the presence of small studies is accompanied by evidence of substantial statistical heterogeneity (I^2 50% with a significant χ^2 statistic).

Analysis of subgroups or subsets

Where the necessary data are available, subgroup analyses will be performed for participant age (child/adult/older adult) and clinical diagnosis; for the type of interaction (passive/active); the setting (public/home aquarium); the presence and type of comparator (no comparator/no treatment control/alternative animal-assisted intervention/other intervention), the length/duration/frequency of exposure; and publication status (published/unpublished).

Contact details for further information

Heather Clements

heather.clements@uws.ac.uk

Organisational affiliation of the review

University of the West of Scotland

www.uws.ac.uk

Review team members and their organisational affiliations

Miss Heather Clements. University of the West of Scotland
Dr Stephanie Valentin. University of the West of Scotland
Dr Nick Jenkins. University of the West of Scotland
Professor Jean Rankin. University of the West of Scotland
Professor Julien Baker. University of the West of Scotland
Dr Donna Snellgrove. WALTHAM Centre for Pet Nutrition
Professor Nancy Gee. WALTHAM Centre for Pet Nutrition; State University of New York, Fredonia
Professor Katherine Sloman. University of the West of Scotland

Anticipated or actual start date

24 January 2018

Anticipated completion date

01 October 2018

Funding sources/sponsors

This systematic review will be conducted as part of the lead author's (HC) PhD which is funded by the University of the West of Scotland and Mars Petcare.

Conflicts of interest

This systematic review will be conducted as part of the first author's (HC) Ph.D., which is partially funded by Mars Petcare. Two authors (DS and NG) are employees of the WALTHAM Centre for Pet Nutrition, a Mars Petcare Research Centre. NG provided feedback on earlier drafts of the protocol, but had no input in the decision to fund the Ph.D. Neither author will be involved in study selection, data extraction or analysis but will provide feedback on the completed review before publication.

Language

(there is not an English language summary)

Country

Scotland, United States of America

Stage of review

Review_Ongoing

Subject index terms status

Subject indexing assigned by CRD

Subject index terms

Fish; Humans; Stress, Psychological

Date of registration in PROSPERO

09 April 2018

Date of publication of this version

09 April 2018

Details of any existing review of the same topic by the same authors

Stage of review at time of this submission

Stage	Started	Completed
Preliminary searches	Yes	Yes
Piloting of the study selection process	Yes	Yes
Formal screening of search results against eligibility criteria	Yes	No
Data extraction	No	No
Risk of bias (quality) assessment	No	No
Data analysis	No	No

Versions

09 April 2018

PROSPERO
This information has been provided by the named contact for this review. CRD has accepted this information in good faith and registered the review in PROSPERO. CRD bears no responsibility or liability for the content of this registration record, any associated files or external websites.
