# Supplementary material for: The effects of interacting with fish in aquariums on human health and well-being: A systematic review
Source: PLoS One. 2019 Jul 29;14(7):e0220524. doi: 10.1371/journal.pone.0220524 (PMC6663029; doi:10.1371/journal.pone.0220524)
Supplement: S3 Appendix — (PDF) [file pone.0220524.s003.pdf]

Reviewer:

Date:

Full study reference:

**General**	
Author/year	
Publication type and source	
Country	
Funding	
Conflict of interest	
**Study details**	
Aim/objectives	
Dates of data collection	
Theoretical framework	
**Methods**	
Study design	
Participant recruitment	
Participants	*Population:*<br>*Age:*<br>*Sex:*<br>*Ethnicity:*<br>*SES:*<br>*Total sample size:*<br>*Sample size per condition:*
Intervention/Independent Variable	
Control	
Allocation to conditions	
Setting/context	
Primary outcomes	*Psychological outcomes (anxiety, depression, behaviour change, social interaction, etc.):*

	Physiological outcomes (heart rate, blood pressure, motor skills etc.):
Secondary outcomes (adverse events, animal welfare, attitudes)	
Brief study description	
**Results**	
Method of analysis	
Primary outcomes	*Psychological outcomes (anxiety, depression, behaviour change, social interaction, etc.):*
	Physiological outcomes (heart rate, blood pressure, motor skills etc.):
Secondary outcomes (adverse events, animal welfare, attitudes)	
**Author conclusions:**	
**Reviewer comments:**	
