# Supplementary material for: The effects of interacting with fish in aquariums on human health and well-being: A systematic review
Source: PLoS One. 2019 Jul 29;14(7):e0220524. doi: 10.1371/journal.pone.0220524 (PMC6663029; doi:10.1371/journal.pone.0220524)
Supplement: S4 Appendix — (PDF) [file pone.0220524.s004.pdf]

S4 Appendix: Quality Appraisal
Qualitative studies

First author and year	Kidd 1999	Langfield 2009
1. Is a qualitative approach appropriate?	Inappropriate<br>Although the title refers to "benefits and problems", which is indicative of a qualitative approach, the design of the questionnaire (open/closed) is unclear. The authors also refer to hypotheses which suggest a quantitative approach	Appropriate<br>Looks at meaning and value participants attribute to keeping pet fish
2. Is the study clear in what it seeks to do?	Unclear<br>The aims are not clearly stated	Clear<br>Aims clearly stated and clear rationale given
3. How defensible/ rigorous is the research design/methodology?	Indefensible<br>The method was not clearly described but appears inappropriate	Not sure<br>Method for recruitment likely to introduce bias
4. How well was the data collection carried out?	Not sure/not reported<br>Data collection is not adequately described	Appropriately<br>Study appears well conducted and methods are robust
5. Is the role of the researcher clearly described?	Not described<br>No information given	Clearly described<br>The relationship of the researcher to participants is clearly explained and a reflective journal was kept throughout
6. Is the context clearly described?	Clear<br>Some information is given about participants and setting, although this could have been developed further	Clear<br>Participant characteristics and context clearly defined, researchers own biases are acknowledged
7. Were the methods reliable?	Unreliable<br>The aims and methods are not adequately described, but the study refers to understanding the benefits and problems of keeping home aquaria - a qualitative approach is therefore appropriate, but the researchers refer to hypothesis testing which is inappropriate	Reliable<br>Data from in-depth interviews, reflective journal and member checking were triangulated. Constant comparison method was used, with two coders involved for at least part of the process
8. Is the data analysis sufficiently rigorous?	Not sure<br>Only descriptive statistics are provided but not clear how these were obtained (i.e. coded from open answers, or closed questionnaire). Analyses performed are however appropriate for descriptive statistics	Not sure<br>Unclear whether a second coder was involved throughout the analytical process, which could have increased rigor

S4 Appendix: Quality Appraisal
Qualitative studies

First author and year	Kidd 1999	Langfield 2009
9. Is the data "rich"?	Poor<br>Only descriptive statistics are presented	Not sure/not reported<br>Some quotes presented but not many, so not clear if these are representative. Only provides perspective of current pet fish owners, other perspectives (i.e. past pet fish owners) could have provided richer data. Does not represent minority groups
10. Is the analysis reliable?	Not sure/not reported<br>Not clear how data were obtained or analysis was conducted, inadequate information	Not sure/not reported<br>Not clear whether two coders were involved in entire process, which could have improved reliability
11. Are the findings convincing?	Not sure<br>There is not enough information about the methods etc. to determine if findings are convincing	Convincing<br>The findings are clear and coherent, evidenced fairly well with extracts/quotes, and are appropriately referenced
12. Are the findings relevant to the aims of the study?	Irrelevant<br>As aims were not stated, it is not possible to know if they were relevant	Relevant<br>They address the aims well
13. Conclusions	Inadequate<br>No specific conclusions are presented	Adequate<br>Conclusions reflect the research findings well
14. How clear and coherent is the reporting of ethics?	Not sure/not reported<br>Unclear whether ethical approval was obtained, reference to "permission" but not informed consent	Appropriate<br>Ethical approval and informed consent were obtained. Although ethics are not discussed in great detail, the study is unlikely to raise major ethical concerns
Overall assessment	-	+

S4 Appendix: Quality Appraisal
Quantitative studies reporting correlation/associations

**First author and year**	**Lin 2013**
1.1 Is the source populations or source area well described?	++<br>Source population was clearly described
1.2 Is the eligible population or area representative of the source population?	+<br>Appears representative but little information given regarding recruitment, beyond being mailed the survey
1.3 Do the selected participants or areas represent the eligible population or area?	+<br>The method of selection was well described, but response rate was only 32.83% so possible self-selection bias. Also unclear whether method of identification (via hospital websites) was appropriate, as these may not have been up to date. Inclusion/exclusion criteria not given
2.1 How was selection bias minimised?	-<br>The response rate was low, so possible self-selection bias. Unclear whether systematic differences exist between those who have/do not have aquariums in workplace (although many confounding factors were controlled)
2.2 Was the selection of explanatory variables based on a sound theoretical basis?	++<br>Clear basis for inclusion of explanatory variables, based on theory and preliminary research
2.3 Was contamination acceptably low?	NA<br>Only one group
2.4 How well were likely confounding factors identified and controlled?	+<br>Adjusted for personal characterisitics, work status and work stres, but did not consider health-related behaviours or pre-existing conditions
2.5 Is the setting applicable to the UK?	+<br>Probably some cultural differences
3.1 Were the outcome measures and procedures reliable?	+<br>Self-report data only, but Cronbach's alpha given for survey questions

S4 Appendix: Quality Appraisal
Quantitative studies reporting correlation/associations

3.2 Were the outcome measurements complete?	-<br>Response rate was low (<40%) and not reported whether all participants completed all questions. However, this is unlikely as there are small discrepancies between the total sample size reported and sample sizes by demographics (e.g. gender, marriage status)
3.3 Were all the important outcomes assessed?	+<br>Outcomes mainly related to self-report of physical health status, but psychological health also likely to be affected by interior amenities
3.4 Was there a similar follow-up time in exposure and comparison groups?	NA<br>Survey design so one time point
3.5 Was follow-up time meaningful?	NA<br>Survey design so one time point
4.1 Was the study sufficiently powered to detect an intervention effect (if one exists)?	NR
4.2 Were multiple explanatory variables considered in the analyses?	+<br>Considered numerous personal and work factors, but some important ones missing, e.g. health-related behaviours, pre-existing medical conditions
4.3 Were the analytical methods appropriate?	+<br>Data analysis was not explained in the methods section. Hierarchical regression can lead to bias due to researcher selecting order in which variables are entered
4.4 Was the precision of association given or calculable? Is association meaningful?	NR<br>Confidence intervals not reported
5.1 Are the study results internally valid (i.e. unbiased)?	-<br>Relied on self-report data which is subjective, likely that other factors could have influenced the outcomes assessed, and bias was introduced by use of hierarchical model
5.2 Are the findings generalizable to the source population (i.e. externally valid)?	+<br>Although all members of thesource population were invited to participate, response rate was low so possibly not representative
Overall study grading	+

S4 Appendix: Quality Appraisal
Quantitative intervention studies

First author and year	Barker 2003	Buttelmann 2014	Cole 2000
1.1 Is the source population or source area well described?	++<br>Population demographics and referral source described well although the location and country should have been stated	-<br>Undergraduate students stated but no more detail provided	+<br>Some description given, but lacking detail
1.2 Is the eligible population or area representative of the source population?	+<br>Recruitment was not well described	+<br>Recruitment was not described	+<br>Recruitment not described and the sample size was too small to be adequate, however it was a pilot study
1.3 Do the selected participants or areas represent the eligible population or area?	+<br>It is not clear how many of the eligible participants agreed to take part, and inclusion/exclusion criteria are not stated	+<br>Selection not described and percentage of potential participants who agreed to take part not given. Inclusion/ exclusion criteria not stated. Over 90% of participants were female	-<br>Selection not well described but appears that participants were selected by the researchers which introduced bias. It is not clear how many of the selected participants agreed to participate. Inclusion/exclusion criteria were given but lacked detail
2.1 How was selection bias minimised?	-<br>Allocation intended to alternate on consecutive appointments, but in reality was determined by service demands	-<br>Participants were placed in groups from baseline measures instead of randomisation	NA<br>No group allocation or cross-over
2.2 Were the interventions (and comparisons) well described and appropriate?	++<br>Interventions and control are described well	++<br>Three interventions described well using descriptions and images	++<br>Aquarium well described and image included, however the type and number of fish used could have been described
2.3 Was the allocation concealed?	NA<br>All participants experienced both conditions	-<br>The person determining allocation strongly influenced the allocation as they determined allocation	NA<br>No group allocation
2.4 Were participants or investigators blind to exposure and comparisons?	+<br>Participants were not aware of the aim of the study, and it appears a researcher who did not collect the data evaluated the data. VAS were rescored by a blind assessor. However, nurses taking measurements were not blinded	-<br>Participants could not be blinded due to the nature of the intervention. It is not clear whether the assessor was blinded, but unlikely	-<br>The participants could not be blinded to the conditions, and it was not reported whether the researchers scoring were blinded, but unlikely

S4 Appendix: Quality Appraisal
Quantitative intervention studies

First author and year	Barker 2003	Buttelmann 2014	Cole 2000
2.5 Was the exposure to the intervention and comparison adequate?	++<br>Exposure time seems appropriate	+<br>Exposure equal in all conditions, but was only 5 minutes, which may not be sufficient	-<br>Longest exposure was eleven days but average time on ward is two months, so not adequate (although this was a pilot)
2.6 Was contamination acceptably low?	+<br>All participants completed both conditions and the wash-out period was not stated, but due to the nature of study contamination is unlikely	+<br>Contamination unlikely but not explicitly stated that participants didn't take part in more than one condition, and as participants were probably known to one another they could have discussed participation	NA<br>Only one condition
2.7 Were other interventions similar in both groups?	NR<br>Not clear whether participants took part in any other treatments which differed across sessions.	++<br>The intervention duration was the same in all conditions. No group received additional interventions.	NA<br>All participants took part in all conditions.
2.8 Were all participants accounted for at study conclusion?	NR<br>Drop out not reported	NR<br>Drop out not reported	NR<br>Drop out not reported
2.9 Did the setting reflect usual UK practice?	++<br>The hospital setting described could be applicable in the UK	++<br>University setting probably reflects UK	++<br>The hospital setting described could be applicable in the UK
2.10 Did the intervention or control comparison reflect usual UK practice?	NA<br>Not a standard intervention	NA<br>University setting	NA<br>Not a standard intervention
3.1 Were outcome measures reliable?	+<br>VAS, HR and BP are standard outcomes. Reports of outcome measure validation from previous studies was included, however VAS may be subjective	+<br>Reliability not explicitly reported for all measures, but likely adequate	++<br>Reliability of the measure was described, based on reports from previous studies

S4 Appendix: Quality Appraisal
Quantitative intervention studies

First author and year	Barker 2003	Buttelmann 2014	Cole 2000
3.2 Were all outcome measures complete?	-<br>Some participants did not complete both conditions so were excluded	-<br>Some participants were excluded because they failed to show anxiety induction	NR<br>Missing data were not reported
3.3 Were all important outcomes assessed?	++<br>Outcomes seemed appropriate and comprehensive	++<br>All outcomes appear to be appropriate	++<br>The outcome measures set seem appropriate to answer the research question
3.4 Were outcomes relevant?	++<br>Outcomes seemed appropriate and set out to measure what was intended	++<br>All outcomes appear relevant	+<br>Some outcome measures seem inappropriate to the research question (e.g. sensation seeking)
3.5 Were there similar follow-up times in exposure and comparison groups?	NA<br>No follow-up	++<br>All groups were treated similarly	NA<br>Only one group
3.6 Was follow-up time meaningful?	NA<br>No follow-up	+<br>A longer follow-up is unlikely to have resulted in differences in outcome, however it was not clear exactly how soon after the intervention the participants were assessed (beyond ‘immediately’)	-<br>Follow-up was eleven days which is probably insufficient
4.1 Were exposure and comparison groups similar at baseline? If not, were these adjusted?	NA<br>All participants completed both conditions	+<br>Authors report that groups were similar at baseline, due to allocation method. However, these data were not reported so cannot be confirmed. Also not clear if outcome measures were similar at baseline	NA<br>All participants in the same group
4.2 Was intention to treat (ITT) analysis conducted?	NA<br>Not relevant to this study design	NA<br>Not relevant to this study design	NA<br>All participants in the same group

S4 Appendix: Quality Appraisal
Quantitative intervention studies

First author and year	Barker 2003	Buttelmann 2014	Cole 2000
4.3 Was the study sufficiently powered to detect an intervention effect (if one exists)?	-<br>Power analyses were conducted on a post hoc basis which is of little value	NR<br>No power analysis reported, although authors acknowledge potential underpowering	NR<br>No power analysis reported
4.4 Were the estimates of effect size given or calculable?	NR<br>Effect sizes were not reported	++<br>Effect sizes were reported for all analyses	NR<br>Effect sizes were not reported
4.5 Were the analytical methods appropriate?	+<br>Some concerns over analysis and whether there were multiple observations per participant, per condition, rather than average scores - this is unclear. However, covariates were pre-specified	-<br>Statistical analysis methods not described. It appears that a one-way ANOVA was performed but this is not appropriate for the multiple groups and multiple time points	-<br>Questionable whether the statistical methods proposed are appropriate. Confounders not adjusted for however the sample size was probably too small for this. Results were not well presented
4.6 Was the precision of intervention effects given or calculable? Were they meaningful?	NR<br>No confidence intervals included	+<br>Confidence intervals presented in graphical form for one analysis, but no values reported	NR<br>No confidence intervals included
5.1 Are the study results internally valid (i.e. unbiased)?	+<br>Some bias has been minimised but allocation was not randomised and nurses were not blinded	-<br>Due to lack of randomisation, this study is heavily biased. Also issues with blinding and statistical analyses	-<br>No control group and lack of blinding are significant biases
5.2 Are the findings generalizable to the source population (i.e. externally valid)?	+<br>Additional participant demographics and inclusion criteria should have been included to make the findings more generalizable	-<br>Participant detail not sufficient to determine if results generalizable. Predominantly female sample likely to skew results	-<br>Sample size too small to be generalizable (although it was a pilot study). Recruitment and selection not well described
Overall study grading	+	-	-

S4 Appendix: Quality Appraisal
Quantitative intervention studies

First author and year	Cracknell 2016	Cracknell 2017 Study 1	Cracknell 2017 Study 2
1.1 Is the source population or source area well described?	-<br>Source population not described beyond ‘students from a University population’	-<br>Poor description beyond "university students"	-<br>Poor description beyond "university students"
1.2 Is the eligible population or area representative of the source population?	+<br>Recruitment and selection not well described	+<br>Recruitment not well described	+<br>Recruitment not well described
1.3 Do the selected participants or areas represent the eligible population or area?	+<br>Selection not described and percentage who agreed to take part not stated. Inclusion/ exclusion criteria not given	+<br>No description of selection process and the percentage of eligible participants who agreed to take part not stated. Some inclusion/exclusion criteria given	+<br>No description of selection process and the percentage of eligible participants who agreed to take part not stated. Some inclusion/exclusion criteria given
2.1 How was selection bias minimised?	-<br>Different participants for each condition at different time points, and participants not randomised (quasi-random)	++<br>Order of photographs was randomised	++<br>Order of photographs was randomised
2.2 Were the interventions (and comparisons) well described and appropriate?	++<br>Excellent description of interventions provided, and stocking information given in supplementary information	++<br>Photographs were described and example images included	++<br>Photographs were described and example images included
2.3 Was the allocation concealed?	-<br>Each condition at a different time point so person determining group allocation could not be concealed to allocation	NA<br>No group allocation	NA<br>No group allocation
2.4 Were participants or investigators blind to exposure and comparisons?	+<br>Participants blind to study aims, but assessors not blinded	+<br>All responses recorded electronically but not possible to blind participants	+<br>All responses recorded electronically but not possible to blind participants

S4 Appendix: Quality Appraisal
Quantitative intervention studies

First author and year	Cracknell 2016	Cracknell 2017 Study 1	Cracknell 2017 Study 2
2.5 Was the exposure to the intervention and comparison adequate?	++<br>Exposure time was consistent across all groups and seemed of adequate duration	+<br>Duration of viewing for each picture not given, but it appears the participants could look at any one image for as long as they liked. Therefore there was no standardisation for the duration of each 'intervention' (image)	+<br>Duration of viewing for each picture not given, but it appears the participants could look at any one image for as long as they liked. Therefore there was no standardisation for the duration of each 'intervention' (image)
2.6 Was contamination acceptably low?	+<br>Contamination unlikely but not explicitly stated that participants didn't take part in more than one condition, and as participants were probably known to each other they could have discussed participation	NA<br>Participants took part in all conditions	NA<br>Participants took part in all conditions
2.7 Were other interventions similar in both groups?	-<br>It is not known whether participants were treated equally in all groups, and authors acknowledged differences in contextual factors across conditions (e.g. weather, number of visitors)	++<br>No other interventions described and due to the short nature of the study duration (one time point) and all participants taking part in all conditions, all can be considered similar per condition	++<br>No other interventions described and due to the short nature of the study duration (one time point) and all participants taking part in all conditions, all can be considered similar per condition
2.8 Were all participants accounted for at study conclusion?	NR<br>Drop out not reported	+<br>There is no report of drop-outs, however participants must have evaluated all images with the nature of the study protocol so it assumed all images were evaluated for those participants	+<br>There is no report of drop-outs, however participants must have evaluated all images with the nature of the study protocol so it assumed all images were evaluated for those participants
2.9 Did the setting reflect usual UK practice?	NA<br>Setting was a National Aquarium so not related to health treatment	NA<br>Not a medical intervention	NA<br>Not a medical intervention
2.10 Did the intervention or control comparison reflect usual UK practice?	NA<br>Setting was a National Aquarium so not related to health treatment	NA<br>Not a medical intervention	NA<br>Not a medical intervention
3.1 Were outcome measures reliable?	++<br>Outcome measures seem appropriate and validity was reported from previous research for some outcome measures	-<br>Subjective outcome measured using a Likert scale without reports of validity	-<br>Subjective outcome measured using a Likert scale without reports of validity

S4 Appendix: Quality Appraisal
Quantitative intervention studies

First author and year	Cracknell 2016	Cracknell 2017 Study 1	Cracknell 2017 Study 2
3.2 Were all outcome measures complete?	+<br>Physiological data missing for some participants so they were excluded from those analyses. All other analyses appear complete	+<br>Missing data not reported but unlikely due to nature of the study	+<br>Missing data were not reported but unlikely due to nature of the study
3.3 Were all important outcomes assessed?	++<br>The important outcomes were assessed	++<br>Outcomes included seem appropriate	++<br>Outcomes included seem appropriate
3.4 Were outcomes relevant?	++<br>The outcomes were relevant	+<br>Outcomes included seem relevant, however validated measures do exist	+<br>Outcomes included seem relevant, however validated measures do exist
3.5 Were there similar follow-up times in exposure and comparison groups?	NA<br>No follow-up	NA<br>No follow-up	NA<br>No follow-up
3.6 Was follow-up time meaningful?	NA<br>No follow-up	NA<br>No follow-up	NA<br>No follow-up
4.1 Were exposure and comparison groups similar at baseline? If not, were these adjusted?	-<br>There were differences in outcome measures at baseline	NA<br>Participants undertook all conditions which were randomised; no baseline assessment	NA<br>Participants undertook all conditions which were randomised; no baseline assessment
4.2 Was intention to treat (ITT) analysis conducted?	NA<br>Not relevant to this study design	NA<br>Only one measurement time point and participants required to rate all images	NA<br>Only one measurement time point and participants required to rate all images

S4 Appendix: Quality Appraisal
Quantitative intervention studies

First author and year	Cracknell 2016	Cracknell 2017 Study 1	Cracknell 2017 Study 2
4.3 Was the study sufficiently powered to detect an intervention effect (if one exists)?	NR<br>No power analysis reported	NR<br>No power analysis reported	NR<br>No power analysis reported
4.4 Were the estimates of effect size given or calculable?	+<br>Effect sizes were reported for some, but not all, outcomes	NR<br>Effect sizes were not reported	NR<br>Effect sizes were not reported
4.5 Were the analytical methods appropriate?	-<br>Data analysis seems incorrect or excessive - mixed ANOVA would be sufficient. Also questionable whether ANOVA should be used for Likert data	+<br>Some of the statistical tests used were incorrect/excessive and a mixed ANOVA is preferable, also questionable whether an ANOVA is appropriate for Likert type data	+<br>Some of the statistical tests used were incorrect/excessive and a mixed ANOVA is preferable, also questionable whether an ANOVA is appropriate for Likert type data
4.6 Was the precision of intervention effects given or calculable? Were they meaningful?	+<br>Confidence intervals provided in graphical form for most outcomes	NR<br>No confidence intervals included	NR<br>No confidence intervals included
5.1 Are the study results internally valid (i.e. unbiased)?	-<br>High likelihood of confounders affecting study outcomes	++<br>Photographs presented in a random order and unlikely to have been influenced by other sources of bias	++<br>Photographs presented in a random order and unlikely to have been influenced by other sources of bias
5.2 Are the findings generalizable to the source population (i.e. externally valid)?	-<br>There is insufficient detail about the study population to identify whether the findings are generalizable	-<br>Insufficient information provided to determine if externally valid	-<br>Insufficient information provided to determine if externally valid
Overall study grading	-	+	+

S4 Appendix: Quality Appraisal
Quantitative intervention studies

First author and year	DeSchriver 1990	Edwards 2002	Edwards 2013
1.1 Is the source population or source area well described?	++<br>Location and setting well described	+<br>Some description but lacking detail	+<br>Some description but lacking detail
1.2 Is the eligible population or area representative of the source population?	+<br>Recruitment methods not stated and no information whether eligible population was representative	+<br>Recruitment was not well described	+<br>Recruitment was not well described
1.3 Do the selected participants or areas represent the eligible population or area?	+<br>Method of selection not stated and not clear what percentage agreed to participate	+<br>Selection was not described and although it is stated that all residents of the home were invited, we do not know what percentage agreed to participate. Inclusion/exclusion criteria not given	+<br>Selection was not described and although it is stated that all residents of the home were invited, we do not know what percentage agreed to participate
2.1 How was selection bias minimised?	+<br>Allocation was randomised but randomisation method not described	-<br>Not clear how the control facility was selected, but unlikely to be random	NA<br>No group allocation or cross-over
2.2 Were the interventions (and comparisons) well described and appropriate?	+<br>Basic detail on the aquarium is provided but more description could have been included	+<br>Basic detail on the aquarium is provided but more description could have been included	+<br>The aquariums were described but more detail is needed, e.g. species
2.3 Was the allocation concealed?	NR<br>Concealment was not reported	NR<br>Concealment was not reported	NA<br>No allocation
2.4 Were participants or investigators blind to exposure and comparisons?	-<br>Neither participants nor assessors were blinded, however this would be impossible due to the nature of the study	-<br>No blinding for the participants or researchers and staff, however this would be difficult to correct for due to the nature of the study	-<br>No blinding for the participants or researchers and staff, however this would be difficult to correct for due to the nature of the study

S4 Appendix: Quality Appraisal
Quantitative intervention studies

First author and year	DeSchriver 1990	Edwards 2002	Edwards 2013
2.5 Was the exposure to the intervention and comparison adequate?	+<br>Exposure was equal across conditions but may have been slightly short	++<br>Exposure time seems appropriate	++<br>Exposure time seems appropriate
2.6 Was contamination acceptably low?	+<br>Contamination during testing was minimised, but all participants could access fish tank between testing sessions	++<br>For the one facility participating in both conditions, a 2-week washout period was used, which seems appropriate	NA<br>There was only one condition
2.7 Were other interventions similar in both groups?	+<br>Duration of interventions the same, but it is not known if any participants in different groups receivied any additional interventions	+<br>The control/treatment group received longer contact with the researchers/programme due to the inclusion of the control phase, however the aquarium time was the same at all facilities	+<br>All participants received same study duration but different staff across each facility could have led to differences in the way participants were treated
2.8 Were all participants accounted for at study conclusion?	NR<br>Drop out not reported	NR<br>Drop out not reported	NR<br>Drop out not reported
2.9 Did the setting reflect usual UK practice?	-<br>Setting unlikely to reflect usual practice as was a purpose built laboratory	+<br>The setting appears to be similar to the UK but likely some local differences	+<br>The setting appears to be similar to the UK but likely some local differences
2.10 Did the intervention or control comparison reflect usual UK practice?	NA<br>Not a standard intervention.	NA<br>Not a standard intervention.	NA<br>Not a standard intervention.
3.1 Were outcome measures reliable?	+<br>All outcome measures seem reliable except the EMG which is known to have poor reliability scores	+<br>Body weight and nutritional intake can be reliable but the methods used to obtain these data were not described	+<br>Measures seem appropriate and staff were trained, but could still be subjective/inaccurate, especially considering environment

S4 Appendix: Quality Appraisal
Quantitative intervention studies

First author and year	DeSchriver 1990	Edwards 2002	Edwards 2013
3.2 Were all outcome measures complete?	NR<br>Missing data were not reported, not clear if all participants completed all time points	+<br>Weight data were reported for all 62 participants but it is not clear if a full set of nutritional intake data are included	+<br>Weight data appears complete but it is not clear if a full set of nutritional intake data are included
3.3 Were all important outcomes assessed?	++<br>All important outcomes assessed	+<br>As data collection was not well described this cannot be known. Also, in the discussion, the researchers refer to additional outcomes of importance (e.g. nutritional supplement use) but did not report whether this was recorded as part of the study	+<br>The authors note that other outcomes (e.g. nutritional supplement use) are important, but these are not assessed
3.4 Were outcomes relevant?	+<br>Most outcomes relevant, with the exception of EMG	+<br>Outcomes assessed appear to be relevant, however this is not clear as description of data collection was inadequate. Also may have been useful to consider quality rather than quantity of food intake	+<br>Outcomes assessed appear to be relevant, although may have been useful to consider quality rather than quantity of food intake
3.5 Were there similar follow-up times in exposure and comparison groups?	NA<br>No follow-up	-<br>The control/treatment group were followed for longer	NA<br>Only one group
3.6 Was follow-up time meaningful?	NA<br>No follow-up	+<br>The time that the aquariums were in place seemed appropriate to detect changes in nutritional status, but perhaps not body mass	+<br>The time that the aquariums were in place seemed appropriate to detect changes in nutritional status, but perhaps not body mass
4.1 Were exposure and comparison groups similar at baseline? If not, were these adjusted?	+<br>All groups were reported to be similar at baseline for age, but while the authors report gender to be similar at baseline, this might be debated. Also differences in outcomes at baseline which were not adjusted for correctly	++<br>No inter-facility differences were found, and all participants took part in the intervention	++<br>There was only one condition and no inter-facility differences were found
4.2 Was intention to treat (ITT) analysis conducted?	NA<br>Not relevant to this study design	+<br>Data on weight were reported for all participants but unclear if nutritional intake data comprises all participants. Also one participant discrepancy in body weight data	+<br>Data on weight were reported for all participants but unclear if nutritional intake data comprises all participants

S4 Appendix: Quality Appraisal
Quantitative intervention studies

First author and year	DeSchriver 1990	Edwards 2002	Edwards 2013
4.3 Was the study sufficiently powered to detect an intervention effect (if one exists)?	NR<br>No power analysis reported	NR<br>No power analysis reported	NR<br>No power analysis reported
4.4 Were the estimates of effect size given or calculable?	NR<br>Effect sizes were not reported	NR<br>Effect sizes were not reported	NR<br>Effect sizes were not reported
4.5 Were the analytical methods appropriate?	-<br>Analysis methods not described so unclear	-<br>No description of the statistical analysis included and didn't appear to account for clustering	-<br>No description of the statistical analysis provided and didn't appear to account for clustering
4.6 Was the precision of intervention effects given or calculable? Were they meaningful?	NR<br>No confidence intervals included	NR<br>No confidence intervals included	+<br>Confidence intervals included in the figures for one analysis only, and no values reported.
5.1 Are the study results internally valid (i.e. unbiased)?	-<br>Potienial bias lack of blinding, and irrelevant EMG, plus poor description of analysis methods so unclear if appropriate	-<br>No blinding and the control group was non-equivalent by design, so findings are based largely on pre-post change from intervention group	-<br>No blinding and the study lacked a control group
5.2 Are the findings generalizable to the source population (i.e. externally valid)?	+<br>More information about the participants and eligible population required	+<br>The sample was well described but unclear whether this is representative of eligible population	+<br>The sample was well described but unclear whether this is representative of eligible population
Overall study grading	-	-	-

S4 Appendix: Quality Appraisal
Quantitative intervention studies

First author and year	Edwards 2014	Katcher 1984	Maranda 2015
1.1 Is the source population or source area well described?	++<br>Setting well described	-<br>No details of the source population provided	++<br>Clinic that participants were recruited from was named, and inclusion/exclusion criteria given
1.2 Is the eligible population or area representative of the source population?	+<br>Recruitment was not well described	+<br>Recruitment method described but little information of the source or sample population	-<br>Recruitment was not well described and unclear whether participants were representative of clinic (source) population
1.3 Do the selected participants or areas represent the eligible population or area?	+<br>Method of selection from the eligible population not described and percentage who agreed to take part not stated. However, inclusion/exclusion criteria were stated	+<br>Selection process not described and percentage of potential participants who agreed to participate was not stated, but inclusion criteria explained	+<br>Selection process not described and percentage of potential participants who agreed to participate was not stated, but inclusion criteria clear
2.1 How was selection bias minimised?	NA<br>No group allocation and no cross-over	+<br>Randomised but method of randomisation not stated	+<br>Randomised but method of randomisation not stated
2.2 Were the interventions (and comparisons) well described and appropriate?	+<br>More detailed description needed	+<br>Intervention procedure was described but no description of the aquarium or poster	+<br>Participants were given same fish bowl and equipment, but purchased their own fish (with funds from researchers), so it is unclear whether they adhered to the instruction to buy a specific species
2.3 Was the allocation concealed?	NA<br>No allocation	NR<br>Concealment was not reported	+<br>States that randomisation sequence was concealed but it is unclear how this was achieved
2.4 Were participants or investigators blind to exposure and comparisons?	-<br>Neither participants nor investigators were blinded, although this was difficult due to the study design	+<br>Assessors were blinded but the participants were not; however blinding participants would not be possible due to the nature of the study	-<br>Impossible to blind participants, and investigators were not blinded either

S4 Appendix: Quality Appraisal
Quantitative intervention studies

First author and year	Edwards 2014	Katcher 1984	Maranda 2015
2.5 Was the exposure to the intervention and comparison adequate?	+<br>Duration of the intervention probably adequate, but perhaps a little too short	++<br>Duration of the intervention seems appropriate	+<br>Length of intervention is appropriate, but unclear how well participants adhered to daily/weekly tasks
2.6 Was contamination acceptably low?	NA<br>There was only one condition	+<br>Not explicitly stated but based on the short-term nature of the intervention this is unlikely	++<br>Authors reported that one participant was excluded due to buying a fish while in control group
2.7 Were other interventions similar in both groups?	+<br>All participants in the same group, however the intervention was delivered across 3 sites and it does not state whether there were any management differences between the sites	-<br>Those in the intervention groups received additional procedures to those in the control group. Although this was part of the study it makes interpretation difficult	++<br>Groups were treated equally apart from planned intervention
2.8 Were all participants accounted for at study conclusion?	++<br>Drop out was reported and acceptable (<10%)	NR<br>Drop out not reported	++<br>Does not appear that any participants were lost to follow-up
2.9 Did the setting reflect usual UK practice?	+<br>The setting appears to be similar to the UK but likely some local differences	++<br>It is unlikely that the setting varied from typical UK dental practice	NA<br>Home setting
2.10 Did the intervention or control comparison reflect usual UK practice?	NA<br>Not a standard intervention	NA<br>Not a standard intervention	NA<br>Not a standard intervention
3.1 Were outcome measures reliable?	+<br>Reliability of the outcomes used was reported, however validity was not stated and not clear why measure was amended	-<br>Methods of physiological data collection and equipment not stated. No comments on validity of the measures, and some outcomes were subjective (dentist ratings of compliance, observer ratings of anxiety)	+<br>Validity and reliability not reported, but measures appear to be used in clinical practice/research

S4 Appendix: Quality Appraisal
Quantitative intervention studies

First author and year	Edwards 2014	Katcher 1984	Maranda 2015
3.2 Were all outcome measures complete?	+<br>Missing data were not explicitly reported but all participants appear to have been included in the analysis	NR<br>Missing data not reported and it is not clear that all participants completed all data points	NR<br>Missing data not reported and it is not clear that all participants completed all data points
3.3 Were all important outcomes assessed?	++<br>All important outcomes appear to have been assessed	++<br>All important outcomes appear to have been assessed	++<br>All important outcomes appear to have been assessed
3.4 Were outcomes relevant?	+<br>Outcomes appear relevant however the reason for adapting existing measures was not explained	++<br>Outcomes assessed appear to be relevant	++<br>Outcomes were relevant
3.5 Were there similar follow-up times in exposure and comparison groups?	NA<br>No follow-up	NA<br>No follow-up	++<br>No differences in follow-up time between groups
3.6 Was follow-up time meaningful?	NA<br>No follow-up	NA<br>No follow-up	++<br>Follow-up seems appropriate
4.1 Were exposure and comparison groups similar at baseline? If not, were these adjusted?	++<br>ANCOVA was used which can adjust for confounders	+<br>Unclear as this was not reported	++<br>Groups were equivalent at baseline
4.2 Was intention to treat (ITT) analysis conducted?	-<br>The one participant who did not complete the study was removed from the dataset	NA<br>Not relevant to this study design	-<br>Only participants with complete data were analysed

S4 Appendix: Quality Appraisal
Quantitative intervention studies

First author and year	Edwards 2014	Katcher 1984	Maranda 2015
4.3 Was the study sufficiently powered to detect an intervention effect (if one exists)?	NR<br>No power analysis reported	NR<br>No power analysis reported	-<br>Power analysis was conducted, but this was poor and so may not be meaningful
4.4 Were the estimates of effect size given or calculable?	NR<br>Effect sizes were not reported	NR<br>Effect sizes were not reported	++<br>Effect sizes were given
4.5 Were the analytical methods appropriate?	-<br>Due to the type of data collected (ordinal), ANCOVA generally not considered appropriate. Clustering was not considered	-<br>Analysis methods not adequately described	-<br>Analysis methods not appropriate, mixed ANOVA should have been used and sub-group analysis specified
4.6 Was the precision of intervention effects given or calculable? Were they meaningful?	NR<br>No confidence intervals included	NR<br>No confidence intervals included	++<br>Confidence intervals reported
5.1 Are the study results internally valid (i.e. unbiased)?	-<br>The study would benefit from a control group and follow-up. Lack of blinding is a significant source of bias, although difficult to control due to study design	+<br>Attempts made to reduce bias by blinding the assessor and randomising. However methods of randomisation not described and unclear whether analysis was appropriate	+<br>Some sources of bias were minimised (e.g. contamination, similarity of groups at baseline,) and there was an attempt at randomisation and allocation concealment, although these are not well described
5.2 Are the findings generalizable to the source population (i.e. externally valid)?	+<br>The sample was well described but unclear whether this is representative of eligible population	-<br>Insufficient information given about the participants to determine whether generalizable	-<br>As a pilot study it is probably not generalizable
Overall study grading	-	-	+

S4 Appendix: Quality Appraisal
Quantitative intervention studies

First author and year	Riddick 1985	Sahrmann 2016	Sanchez 2015
1.1 Is the source population or source area well described?	++<br>Source population described in detail	+<br>Some description of the source population but more detail beneficial	-<br>Poor description of the source population
1.2 Is the eligible population or area representative of the source population?	-<br>Recruitment methods described, however control group participants identified by the centre manager, hence considerable selection bias	+<br>Recruitment and selection methods described but may not be representative as only adults measured, hence unlikely to be representative of the entire source population	+<br>Recruitment methods not described and not clear if representative
1.3 Do the selected participants or areas represent the eligible population or area?	-<br>Methods of selection from eligible participants not described except for the control group which was hand-picked by the centre manager. Percentage that agreed to take part included, and inclusion/ exclusion criteria stated	+<br>The researchers refer to previous (albeit unpublished) research which examined the typical characteristics of visitors to the exhibit; the selected participants matched the eligible population on characteristics such as gender. However, not clear what percentage of the eligible population took part	+<br>Eligible population not described and method of selection not stated, but some inclusion/exclusion criteria given
2.1 How was selection bias minimised?	-<br>Majority of participants in intervention groups chose their preferred group, and control group was handpicked by centre manager, so considerable bias	NA<br>There was only one condition	-<br>Control group were randomly selected from those participating in the intervention group, but method of selection is not stated and order of interventions was not randomised
2.2 Were the interventions (and comparisons) well described and appropriate?	+<br>Some description of the aquarium but more detail would have been beneficial	+<br>Intervention mostly well described but some additional information beneficial, e.g. stocking	++<br>Intervention described well and images were included to support this
2.3 Was the allocation concealed?	-<br>Concealment unlikely as allocation was mostly by preference	NA<br>No allocation	NR<br>Concealment was not reported
2.4 Were participants or investigators blind to exposure and comparisons?	-<br>Participants and assessors not blinded, but this would be hard to achieve due to design of study	-<br>Participants and assessors not blinded, but this would be hard to achieve due to design of study	-<br>Participants not blinded and unclear if researchers/ assessors were blinded

S4 Appendix: Quality Appraisal
Quantitative intervention studies

First author and year	Riddick 1985	Sahrmann 2016	Sanchez 2015
2.5 Was the exposure to the intervention and comparison adequate?	++<br>Exposure was appropriate	+<br>The exposure was probably adequate, however 10 minutes is not representative of average visit time (20 mins)	++<br>Duration of exposure adequate and equal between conditions
2.6 Was contamination acceptably low?	-<br>It is not known if contamination occurred, as residents in the complex could have visited someone with a fish tank	NA<br>There was only one condition	-<br>The control group had previously participated in the intervention group, so were already familiar with the procedure - contamination likely
2.7 Were other interventions similar in both groups?	+<br>Participants in each group were treated similarly by researchers, but not clear if other interventions were similar across groups	NA<br>There was only one condition	-<br>The control group were followed for longer as they had already participated in the intervention group
2.8 Were all participants accounted for at study conclusion?	+<br>Drop out was reported and acceptable (<10%) but all from control group	NR<br>Drop out not reported	NR<br>Drop out not reported
2.9 Did the setting reflect usual UK practice?	+<br>Likely to be some differences due to age and location of study	NA<br>Public aquarium setting	NA<br>Not a standard intervention
2.10 Did the intervention or control comparison reflect usual UK practice?	NA<br>Not a standard intervention	NA<br>Public aquarium setting	NA<br>Not a standard intervention
3.1 Were outcome measures reliable?	+<br>Measures obtained have reliability and validity reports but psychological assessments were interview administered which may have introduced bias	+<br>No reports of reliability but physiological methods are generally reliable and psychological assessment has been validated	+<br>Authors state that the pain assessment method is highly reliable, but no data given

S4 Appendix: Quality Appraisal
Quantitative intervention studies

First author and year	Riddick 1985	Sahrmann 2016	Sanchez 2015
3.2 Were all outcome measures complete?	NR<br>Missing data were not reported	+<br>Physiological data were missing due to technical problems, but these were excluded. Not explicitly stated that all other measures are complete	NR<br>Missing data were not reported and not clear whether all participants completed all time points
3.3 Were all important outcomes assessed?	++<br>All important outcomes appear to have been assessed	++<br>Outcome measures obtained seem appropriate	-<br>Other parameters such as heart rate could have been measured
3.4 Were outcomes relevant?	++<br>Outcomes were relevant	++<br>Outcomes were relevant	+<br>The outcomes used were relevant, but not in isolation
3.5 Were there similar follow-up times in exposure and comparison groups?	NA<br>No follow-up	NA<br>No follow-up	-<br>Control group 'followed' for longer as they participated in both conditions
3.6 Was follow-up time meaningful?	NA<br>No follow-up	NA<br>No follow-up	++<br>Duration of follow-up is appropriate for this study
4.1 Were exposure and comparison groups similar at baseline? If not, were these adjusted?	-<br>Differences between groups at baseline were not adjusted for. Also differences due to allocation by preference	NA<br>There was only one condition	-<br>Unclear as not reported, but control group was a small subset of the intervention group so unlikely that they were similar
4.2 Was intention to treat (ITT) analysis conducted?	-<br>Participants who dropped out were not included in analysis	NA<br>Not relevant to this study design	NA<br>Not relevant to this study design

S4 Appendix: Quality Appraisal
Quantitative intervention studies

First author and year	Riddick 1985	Sahrmann 2016	Sanchez 2015
4.3 Was the study sufficiently powered to detect an intervention effect (if one exists)?	- No power analysis reported, and methods used to account for being underpowered are inappropriate	NR No power analysis reported	NR No power analysis reported
4.4 Were the estimates of effect size given or calculable?	NR Effect sizes were not reported	NR Effect sizes were not reported	NR Effect sizes were not reported
4.5 Were the analytical methods appropriate?	- Statistical analysis not appropriate and poorly reported	++ Appropriate statistical analysis	- Analysis methods not appropriate due to use of multiple tests rather than omnibus test
4.6 Was the precision of intervention effects given or calculable? Were they meaningful?	NR No confidence intervals included	+ Some confidence intervals stated in the figures but no numerical values provided	NR No confidence intervals included
5.1 Are the study results internally valid (i.e. unbiased)?	- Considerable flaws in study design, particularly with regards to selection bias	- Well reported but bias present due to lack of blinding and the study would benefit from a control group	- Main flaws were the inappropriate control group and lack of blinding
5.2 Are the findings generalizable to the source population (i.e. externally valid)?	- Recruitment well described but selection process unclear, with exception of control group who were handpicked by centre manager	+ Recruitment and selection fairly well described, but more detail could have been given about participants	- Recruitment and selection not well described and more details regarding sample needed. Also study is investigating an intervention for children, and although ethical issues prevent using child participants for this preliminary research, the findings can't be generalized to children
Overall study grading	-	-	-

S4 Appendix: Quality Appraisal
Quantitative intervention studies

First author and year	Wells 2005
1.1 Is the source population or source area well described?	+<br>Some information of the setting given but the source population would benefit from more description
1.2 Is the eligible population or area representative of the source population?	+<br>Recruitment was not well-described
1.3 Do the selected participants or areas represent the eligible population or area?	+<br>Eligible population not described and method of selection not stated. However, the inclusion/exclusion criteria were given
2.1 How was selection bias minimised?	+<br>Randomised but method of randomisation not stated
2.2 Were the interventions (and comparisons) well described and appropriate?	++<br>Fairly well explained and comparisons appropriate
2.3 Was the allocation concealed?	NR<br>Concealment was not reported
2.4 Were participants or investigators blind to exposure and comparisons?	+<br>Participants nor researchers blinded to conditions, although participants were not aware of study aims or other conditions

S4 Appendix: Quality Appraisal
Quantitative intervention studies

First author and year	Wells 2005
2.5 Was the exposure to the intervention and comparison adequate?	++<br>Duration of exposure adequate and equal between conditions
2.6 Was contamination acceptably low?	+<br>Contamination unlikely but not explicitly stated that participants didn't take part in more than one condition, and as participants were probably known to one another they could have discussed participation
2.7 Were other interventions similar in both groups?	++<br>Interventions were similar and same procedure was used
2.8 Were all participants accounted for at study conclusion?	NR<br>Drop out not reported
2.9 Did the setting reflect usual UK practice?	NA<br>University setting
2.10 Did the intervention or control comparison reflect usual UK practice?	NA<br>University setting
3.1 Were outcome measures reliable?	+<br>Outcomes generally reliable, although the specific reliability of the devices used was not stated

S4 Appendix: Quality Appraisal
Quantitative intervention studies

First author and year	Wells 2005
3.2 Were all outcome measures complete?	NR<br>Missing data were not reported and not clear whether all participants completed all time points
3.3 Were all important outcomes assessed?	+<br>Would have been beneficial to measure some psychological outcomes
3.4 Were outcomes relevant?	++<br>Outcomes were relevant
3.5 Were there similar follow-up times in exposure and comparison groups?	NA<br>No follow-up
3.6 Was follow-up time meaningful?	NA<br>No follow-up
4.1 Were exposure and comparison groups similar at baseline? If not, were these adjusted?	+<br>Unclear as not described. Also a discrepancy as authors state 10 male and 10 female participants were assigned to each group but also that 58% were male
4.2 Was intention to treat (ITT) analysis conducted?	NA<br>Not relevant to this study design

S4 Appendix: Quality Appraisal
Quantitative intervention studies

First author and year	Wells 2005
4.3 Was the study sufficiently powered to detect an intervention effect (if one exists)?	NR<br>No power analysis reported
4.4 Were the estimates of effect size given or calculable?	NR<br>Effect sizes were not reported
4.5 Were the analytical methods appropriate?	++<br>Analysis methods appropriate
4.6 Was the precision of intervention effects given or calculable? Were they meaningful?	NR<br>No confidence intervals included
5.1 Are the study results internally valid (i.e. unbiased)?	+<br>Overall well designed but some bias still present due to lack of blinding
5.2 Are the findings generalizable to the source population (i.e. externally valid)?	+<br>Likely to be generalisable to source population, but more information is needed
Overall study grading	+
